# An evaluation of the replicability of analyses using synthetic health data

**DOI:** 10.1038/s41598-024-57207-7

**Published:** 2024-03-24

**Authors:** Khaled El Emam, Lucy Mosquera, Xi Fang, Alaa El-Hussuna

**Affiliations:** 1 School of Epidemiology and Public Health, University of Ottawa, Ottawa, ON Canada; 2 Replica Analytics, Ottawa, ON Canada; 3 Children’s Hospital of Eastern Ontario (CHEO) Research Institute, 401 Smyth Road, Ottawa, ON K1H 8L1 Canada; 4 OpenSourceResearch, Aalborg, Denmark

**Keywords:** Bioinformatics, Information technology, Epidemiology

## Abstract

Synthetic data generation is being increasingly used as a privacy preserving approach for sharing health data. In addition to protecting privacy, it is important to ensure that generated data has high utility. A common way to assess utility is the ability of synthetic data to replicate results from the real data. Replicability has been defined using two criteria: (a) replicate the results of the analyses on real data, and (b) ensure valid population inferences from the synthetic data. A simulation study using three heterogeneous real-world datasets evaluated the replicability of logistic regression workloads. Eight replicability metrics were evaluated: decision agreement, estimate agreement, standardized difference, confidence interval overlap, bias, confidence interval coverage, statistical power, and precision (empirical SE). The analysis of synthetic data used a multiple imputation approach whereby up to 20 datasets were generated and the fitted logistic regression models were combined using combining rules for fully synthetic datasets. The effects of synthetic data amplification were evaluated, and two types of generative models were used: sequential synthesis using boosted decision trees and a generative adversarial network (GAN). Privacy risk was evaluated using a membership disclosure metric. For sequential synthesis, adjusted model parameters after combining at least ten synthetic datasets gave high decision and estimate agreement, low standardized difference, as well as high confidence interval overlap, low bias, the confidence interval had nominal coverage, and power close to the nominal level. Amplification had only a marginal benefit. Confidence interval coverage from a single synthetic dataset without applying combining rules were erroneous, and statistical power, as expected, was artificially inflated when amplification was used. Sequential synthesis performed considerably better than the GAN across multiple datasets. Membership disclosure risk was low for all datasets and models. For replicable results, the statistical analysis of fully synthetic data should be based on at least ten generated datasets of the same size as the original whose analyses results are combined. Analysis results from synthetic data without applying combining rules can be misleading. Replicability results are dependent on the type of generative model used, with our study suggesting that sequential synthesis has good replicability characteristics for common health research workloads.

## Introduction

There has been growing interest in using synthetic data generation (SDG) techniques to enable broader sharing of health data for research and analysis^1–11^, and SDG has been highlighted as a key privacy enhancing technology for data access in the coming decade^12^. Furthermore, there are recent examples of health research studies using synthetic data not requiring ethics approval because they are considered to contain no patient information^13^, which can greatly accelerate research projects.

There are multiple synthetic health datasets that are being made available to a broad research community such as: the NIH National COVID Cohort Collaborative (N3C)^14^, the CMS Data Entrepreneur’s Synthetic Public Use files^15^, synthetic cardiovascular and COVID-19 datasets available from the CPRD in the UK^16,17^, A&E data from NHS England^18^, a synthetic dataset from the Dutch cancer registry^19^, cancer data from Public Health England^20^, synthetic variants of the French public health system claims and hospital dataset (SNDS)^21^, and the South Korean data from the Health Insurance Review and Assessment service (the national health insurer)^22^. Furthermore, recently authors have been making synthetic variants of data used in their research papers publicly available^23^, to enable open science.

An important criterion for evaluating synthetic data is its utility. Utility is assessed by the data custodian before sharing the synthetic data with the eventual data users. The eventual data users would only have access to the synthetic data and not to the real datasets that were used to train the generative models.

Utility metrics can be defined as broad or narrow^24^. Broad metrics are generic and do not take into account the specific analytic workloads that the synthetic dataset will be used for^25^. Most of these metrics focus on the fidelity of the synthetic data to the real data by assessing the similarity of the joint distributions of both datasets. They are useful, for example, to compare and improve SDG methods^26–28^. Narrow metrics are specific to an analysis that is performed with synthetic data. They are also sometimes referred to as workload-aware utility metrics. The data custodian would often not have a precise knowledge of individual user workloads in advance, and therefore utility is evaluated on commonly used workloads instead. Our focus in this study is on these narrow metrics.

One definition of narrow utility is *replicability*. Replicability is the reliability of findings when an existing study is repeated using the same analytical methods but different data^29^. There are two interpretations of replicability in the context of SDG.

**Figure 1:**
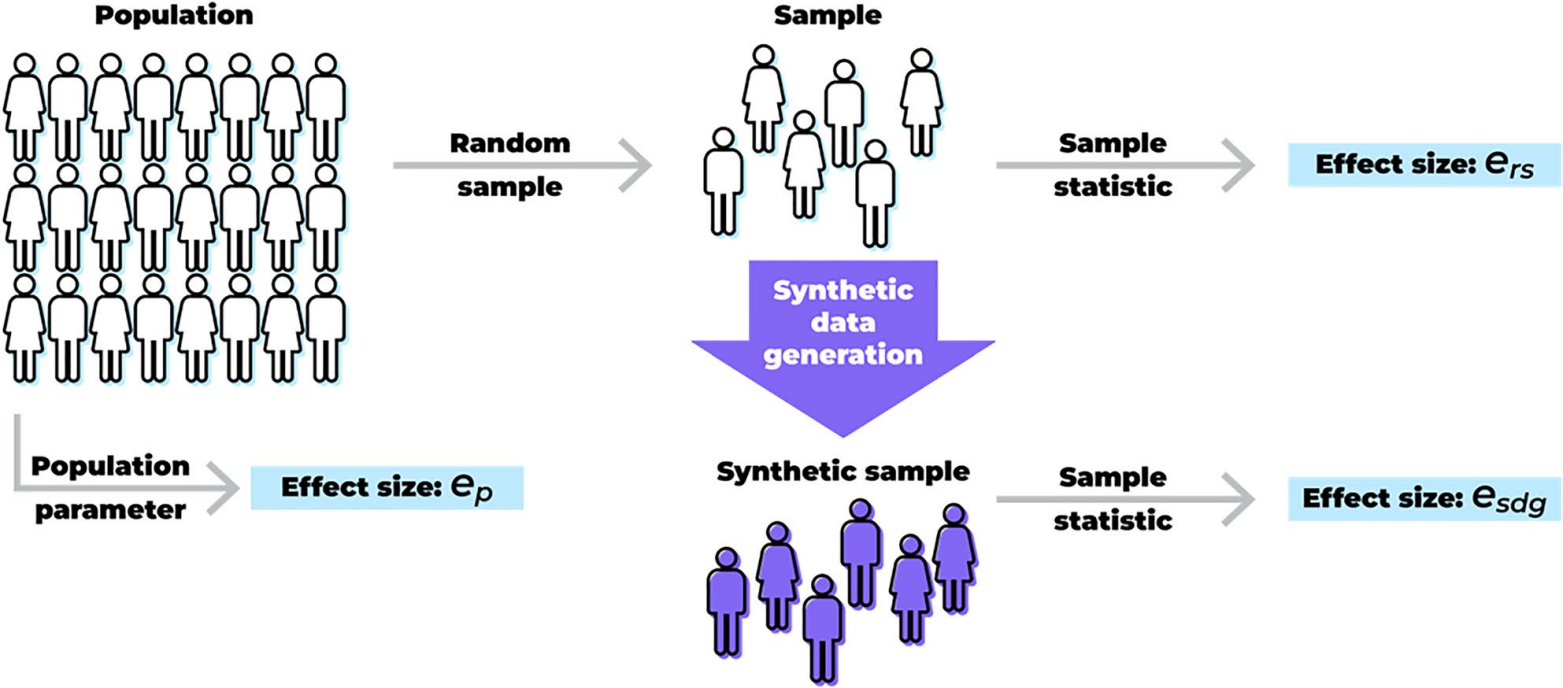
Different approaches for evaluating the “narrow” utility of synthetic data in terms of replicability.

Under one interpretation, replicability is assessed by comparing the analysis results using the real datasets with the results of the same analysis performed on the synthetic data, and is illustrated in Fig. 1. Here the effect size from a specific real dataset, which is a sample from some population, is computed and denoted by $e_{rs}$, then $e_{rs}$ is compared to the parameter estimate from the synthetic data, $e_{sdg}$, for example, by evaluating the confidence interval overlap^24^. It is quite common to evaluate the utility of SDG techniques using this approach^1–11^. In the current study we define objective criteria for such an evaluation.

Another interpretation of replicability is whether population inferences made using synthetic data are valid^30^. In this case the comparison is between $e_{sdg}$ and the population value of the parameter, $e_{p}$. For this type of utility evaluation, standard metrics such as bias, coverage, precision, and statistical power become more relevant^31^.

The original proposal for SDG treated it as a form of multiple imputation^32^. Under the multiple imputation model, multiple datasets, say *m* , are synthesized and combining rules are used to compute the parameter estimates and variances across the *m* synthetic datasets^33,34^. Additional variance adjustment and combining rules were introduced for singly imputed synthetic data (i.e., *m* = 1)^35^. Such corrections ensured that variability introduced by the synthesis process are accounted for when computing parameter estimates, their standard errors, and making population inferences from synthetic datasets.

Disclosing *m* synthetic datasets to the data analysts could also increase the privacy risks. While synthetic data is deemed to have low identity disclosure risks in practice because there is not a one-to-one mapping between synthetic records and real people^36–43^, it still has other types of disclosure risks, such as membership disclosure^44–47^. Therefore, it is important to evaluate the privacy implications when generating and sharing *m* synthetic datasets.

Previous studies evaluating the effect of the combining rules on analysis results from synthetic data used simulated datasets that were not specific to health data^35^, performed more qualitative evaluations of study results^48,49^, or focused primarily on disclosure risks^39^. These studies did not provide a set of specific recommendations for the application of the multiple imputation combining rules for health data, and did not consider both types of replicability criteria^30^: (a) the similarity of analysis findings to those from real data, and (b) the validity of population inferences.

In this paper we therefore perform a simulation study to evaluate the two types of replicability criteria, and also answer the following questions:

**Table:** 

Q1	How many synthetic datasets should be generated and combined (i.e., what is the appropriate value of *m*) to maximize the replicability of results using SDG ? The values of m varied from 1 to 500 in previous work^35,43,48,50–52^. There has not been a comprehensive assessment of the appropriate number of synthetic datasets to be generated
Q2	What are the privacy risks from sharing *m* synthetic datasets? There has been limited research on the privacy risks when multiple synthetic datasets from the same real dataset are released
Q3	Would the amplification of the synthetic datasets improve the replicability of SDG results ? A naïve amplification whereby synthetic data is larger than the real data will result in an inflation of statistical power, however, how will this amplification affect both replicability criteria with the application of combining rules ?
Q4	What are the differences in the performance of two of the more common SDG methods, sequential synthesis and generative adversarial networks, with respect to the replicability of analysis results using the generated datasets ?

Our results addressing these questions can inform how well common SDG methods enable replication of analyses using synthetic data, and the overall evaluation approach can be used in future utility benchmarking studies.

## Methods

We present the simulation design in the ADEMP format as recommended for simulation studies by Morris et al.^31^.

### Aim

The aim of this simulation study was to evaluate the replicability of common statistical analyses performed on synthetic data, and answer the four questions in the introduction about various factors that may impact replicability.

### Data generating mechanisms

#### Simulating a population

To perform the Monte Carlo simulations, we need to have a population of patients, which we then sample from. There are multiple approaches to simulating a population. One can define distributions of convenience (e.g., Gaussian) for a number of variables and sample from those, define a regression model with arbitrary effect sizes, and use the latter to generate outcome variables^50^. This general approach produces a population that is not grounded in realistic health data, and typically treats the predictor variables as independent, which is an assumption that is unlikely to be true in practice. We instead use an approach that is common in health data simulations, whereby we start from real datasets and then we sample with replacement to generate simulated samples^31,53^.

#### Datasets

The three health datasets evaluated in this simulation study covered multiple conditions, jurisdictions, and data collection approaches, as summarized in Table 1. More details about these datasets are included in the supplementary materials.

**Table 1:** The three datasets and their characteristics.

Dataset	Population	Sample size^a^	Model	Event rate	True effect size (odds ratio)^b^
N0147	1,365,135	1420	Impact of bowel obstruction at presentation on 5 year survival	0.12	1.8
CCHS	35.44 m	903	Impact of sex on cardiovascular health	0.63	1.47
DCCG	30,000	2625	Impact of sex on medical complications after right colon resection surgery	0.16	1.58

^a^At 80% power.

^b^For parameter of interest.

The first was the control arm from a colon cancer clinical trial (N0147 trial)^54,55^. The second dataset was the 2014 Canadian Community Health Survey (CCHS), which is conducted by Statistics Canada and represents the population of Canada. The third dataset was a prospectively maintained Danish Colorectal Cancer Group (DCCG) database including all Danish patients with a first-time diagnosis of right-sided colonic cancer between 2001 and 2018^56^.

The analytic workload was logistic regression (LR). For each dataset a specific parameter was of interest and that was the focus of our simulations. More details on the parameter of interest and the LR model covariates for each dataset are provided in the supplementary materials.

According to a classification of odds ratio effect sizes^57^, the effect sizes for the parameter of interest in N0147, CCHS, and DCCG are all small (defined as OR between 1.28 and 1.8) with the largest OR at 1.8 for the N0147 dataset. This is slightly smaller than the median OR in epidemiological studies of 2.16. However, published studies tend to have effect sizes biased upwards compared to all analyses that are conducted^58,59^. Therefore, the effect sizes we simulate are arguably quite close to the median ones in health research and representative of current research.

#### Dataset sample size

The sample size for the datasets used in the simulations was that deemed sufficient to achieve an 80% power for the LR parameter of interest using the true effect size, correlations, and event rate based on standard power equations^60,61^. Because real data do not satisfy all of the assumptions, this calculated value was used as a floor. We then performed a Monte Carlo simulation with 1000 iterations using that calculated sample size as the starting point to determine the empirical 80% power sample size, which was used in our studies. For example, if the calculated sample size using the power equations was 100 observations, we then sampled 1000 datasets from the population we created of 100 observations each and computed the empirical power. If that was below 80% then the simulation was re-run with 110 observations, and so on, incrementing the sample size until the 80% power was reached. The sample size that achieves 80% power is the one shown in Table 1.

#### Study design

We followed a fully factorial design with the following factors considered: generative model (two types of generative models), whether to adjust for multiple synthetic datasets (Y/N), number of synthetic datasets that are generated ($m$, the number of datasets, varied from 1 to 20), and number of different data amplifications (4 levels). This provides 320 different scenarios for each of the three datasets considered.

### Target of analysis

#### Analytic workload

We used LR models because they are common in health research for diagnostic and prognostic modeling^62^. A recent systematic review has shown that LR performance is comparable to the use of machine learning models for clinical prediction workloads^63^. Furthermore, an evaluation of the relative accuracy of LR models compared to other machine learning techniques, such as random forests and SVM, on synthetic versus real datasets across multiple types of SDG methods showed that LR models are only very slightly different^64^. Therefore, evaluating LR model parameters would have broad applicability for health research.

#### Estimand

A different model was fit for each dataset. The specific estimand of interest is described below in the context of the LR model. For our analysis the Wald confidence interval was computed.

For the N0147 dataset, we evaluated the impact of bowel obstruction on 5 year survival as a binary outcome^65^. The CCHS model we constructed evaluated cardiovascular health using the CANHEART index^66^, which was dichotomized at the “poor” to “intermediate” health boundary, and the covariate of interest was sex^67^. The DCCG model we constructed examines the relationship between sex and medical complications^68,69^.

#### Adjustment using multiple imputation combining rules

Assume that we are estimating a particular model parameter of interest *q*_*i*_ with variance *v*_*i*_ using synthetic dataset *i* where *i* = 1 … *m*. The adjustment for the model parameters and variances are as follows^35^. The combined model parameter $\overline{q}_{m}$ is the mean across the *m* model parameters from the synthetic datasets $\overline{q}_{m} = 1/m\sum\nolimits_{i} {q_{i} }$ , and $\overline{v}_{m}$ is the mean variance across the *m* model parameters from the synthetic datasets $\overline{v}_{m} = 1/m\sum\nolimits_{i} {v_{i} }$. The adjusted variance is computed as *T*_*f*_ = $\overline{v}_{m}(k/n + 1/m)$ where *k* is the size of the synthetic dataset and *n* is the size of the real dataset, and the adjusted large sample 95% confidence interval of the model parameter is computed as $\overline{q}_{m}$ ± 1.96 $\sqrt {T_{f} }$.

This means that as the value of *k* increases above *n* the adjusted variance will also increase. This will have an impact on inferential validity, and imposes a cost to data amplification through synthesis.

Note that even with a single synthetic dataset with no amplification, $1/m = 1$ in the combining rules, therefore the parameter CI width is still increased by $\sqrt 2$ under the multiple imputation approach. This means that the CI for a model from a single synthetic dataset and from a single synthetic dataset with the combining rules applied are not the same.

### Methods (generative models evaluated)

We used two types of generative models: a sequential synthesis model and a generative adversarial network (GAN). These two types of generative models are representative of those used in practice. Sequential synthesis using decision trees was one of the first machine learning approaches proposed in the literature^70,71^ and has since been used extensively to synthesize health and social sciences data^35,71–78^, and applied in research studies on synthetic data^48,71,79^. More recently GANs have been one of the more used types of generative models in research and practice^80–82^, and have been applied often for the synthesis of health data^37,44,46,83–85^.

#### Overview of sequential synthesis

The first type of generative models was a sequential decision tree-based synthesizer^28^. Each model in the sequence was trained using a gradient-boosted decision tree algorithm^86,87^, with Bayesian optimization and fivefold cross-validation for hyperparameter tuning^88^. The variable sequence is optimized using a particle swarm algorithm^28^.

**Figure 2:**
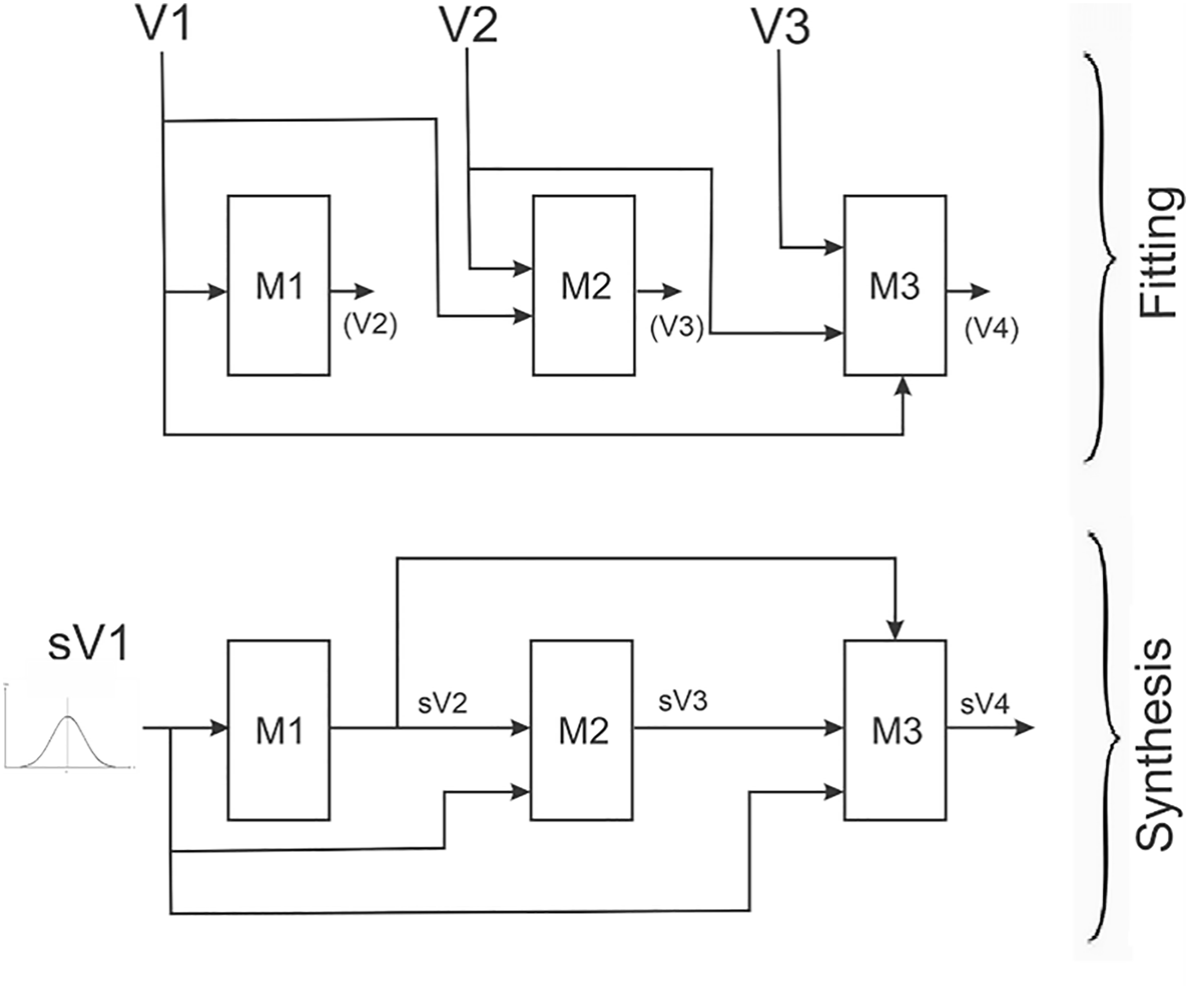
Illustration of the sequential synthesis process for a four-variable dataset.

The process of sequential synthesis is illustrated in Fig. 2 for a four-variable dataset: V1 to V4. In the fitting phase, three models are constructed: M1 to M3. As shown, the first model takes as input V1 and produces V2 as the outcome. The nature of the variables, whether categorical or continuous, does not affect the process, as the model adjusts to become either a classification tree or a regression tree accordingly. The second model in the sequence takes V1 and V2 as input with V3 as the outcome, and so on.

The synthesis step is initiated by sampling from the actual or fitted distribution of the first variable, V1. This creates the synthetic version of that variable sV1. Sampled values are then entered into the first model to generate the distribution of sV2. The synthetic value of sV2 is either sampled according to the predicted probabilities (for categorical variables) or smoothed using a kernel density estimator with boundary correction (for continuous variables)^89^, with bandwidth computed from the original data.

Having generated two synthetic values, sV1 and sV2, these form the input for model M2 to produce the distribution of sV3. Again, the generated synthetic value is either sampled from that predicted distribution or smoothed. The process proceeds in that manner until all variables are synthesized.

#### Overview of GAN

The second type of generative model is CTGAN^90^, which is a conditional GAN architecture.

In its basic form, a vanilla GAN consists of two multi-layer-perceptron neural networks, viz., a generator and a discriminator. The generator and the discriminator play a min–max game. The input to the generator is noise while its output is synthetic data. The discriminator has two inputs: the real training data and the synthetic data generated by the generator. The output of the discriminator indicates whether its input is real or synthetic. The generator is trained to ‘trick’ the discriminator by generating samples that look real. On the other hand, the discriminator is trained to maximize its discriminatory capability.

There are many variations of the vanilla GAN that are widely used for different applications. For instance, Bourou^91^ provides a review of GANs used in tabular data synthesis. Conditional GAN was first introduced by Mirza^92^. Of special interest is the CTGAN proposed by Xu^93^. CTGAN was developed to tackle several challenges when modelling tabular data. Among these are the multimodal distributions of continuous variables and highly imbalanced categorical variables. CTGAN, solves the first problem by proposing a per-mode normalization technique. The second problem is solved by a conditional GAN where each category of a categorical variable serves as the condition passed to the GAN.

### Performance measures

#### Replicability metrics

The performance measures that were used to evaluate replicability are summarized in Table 2 (to evaluate replicability defined as the ability to draw the same conclusions as the analysis on the real dataset^94^) and Table 3 (to evaluate replicability defined as the validity of population inferences from the synthetic datasets^31^).

**Table 2:** The definitions of the metrics that were used to evaluate replicability defined as the ability to draw the same conclusions as the analysis on the real data^94^.

Metric	Interpretation
Decision agreement	A Boolean indicator of whether the same conclusion is drawn from the real and synthetic estimates. This means that the synthetic data estimates have the same direction and statistical significance as the real data. The decision agreement does not apply if the analysis is descriptive. This is consistent with previous measures of replicability^105–107^, has been used to compare real world data analysis results against a clinical trial reference^108–112^, and to assess the replicability of psychological studies^107^. Decision agreement is computed as the proportion across all 1000 simulation runs. We would expect this to be equal to power, which is 80%^108^
Estimate agreement	A Boolean indicator of whether the estimate produced by the synthetic data is within the 95% CI produced by the real data. This requires that a synthetic data effect estimate be within the range of plausible values for the true effect based on evidence from the real data. This is consistent with previous measures of replicability^106,107,113^, has been used to compare real world data analysis results against a clinical trial reference^108–112^, and to assess the replicability of psychological studies^107^. Estimate agreement is computed as the proportion across all 1000 simulation runs. Under the assumption that the parameter variances are equal between the real and synthetic datasets, the expected estimate agreement is 83% under no bias^108^
Standardized difference	A Boolean indicator of whether the difference in the parameter estimate is consistent with the null hypothesis of no difference^108^. The Z value is computed and compared to the standard normal (|Z|< 1.96). The expected value is that this would be at least 95% across all 1000 simulation runs
CI overlap	The proportion of the real and synthetic CIs overlap^24^, which is a commonly used SDG utility metric. This is averaged across all 1000 simulation runs. We would want this to be as close to 100% as possible

**Table 3:** The definitions of the metrics that were used to evaluate replicability defined as the validity of population inferences from the synthetic datasets^31^.

Metric	Interpretation
Bias	The difference between the parameter estimate averaged across all the simulation runs and the true value in the population. We would want this to be as close to zero as possible
Bias-eliminated coverage	The proportion of 95% confidence intervals that include the average parameter estimate across all simulation iterations. We would want this to be at 0.95
Power	The proportion of simulation iterations where the parameter estimate is statistically significant. We would want this to be as close to 80%, or higher
EmpSE	The empirical standard deviation of the parameter estimate from the empirical average, averaged across all simulation runs. It is a measure of the precision of the parameter estimate across runs. We would want this to be as small as possible
Privacy	The membership disclosure metric computed on the pooled datasets for that value of m^95^. The acceptable threshold for this relative F1 score metric is 0.2^67,94,95^

#### Privacy metric—membership disclosure

Privacy risks were computed using a membership disclosure metric^95^. Membership disclosure evaluates the ability of an adversary to correctly determine if a target individual is in the original data that was used to train the generative model. The metric is a relative F1 score that evaluates the accuracy of such adversary attacks compared to a naïve attack which does not use the information in the synthetic data. Previous work has used a threshold of 0.2 to determine if the relative F1 score was low enough^67,94,95^.

Membership disclosure was evaluated by pooling all of the $m$ synthetic datasets. Although in practice we did not observe a difference in the membership disclosure risk between the $m$ pooled datasets or when evaluating a single dataset. The results shown consider the $m$ pooled datasets.

To compute the membership disclosure risk we need to have a measure of the population size. For the colon cancer dataset there were 1,365,135 people living with colorectal cancer in the US in 2018^96^, which we set as our population size. For the CCHS dataset we used the population of Canada in 2014 since that was a population survey. The prevalence of colon cancer in Denmark is approximately 30,000^97^. These values are summarized in Table 1.

Membership disclosure is different from identity disclosure (commonly referred to as re-identification risk), in that a dataset can have a low re-identification risk but still have a high membership disclosure risk. Although the original datasets that were used in this study were deemed to be de-identified already, it is still necessary to assess membership disclosure risk.

#### Number of simulations

The number of simulation iterations was set to 1000 for each simulated scenario. This is the most common value for the number of simulation iterations used in the medical statistics literature^31,98^. This is also consistent with assuming a Monte Carlo standard error of 0.7% for a 95% CI coverage evaluation^31^, which is a key performance parameter in our study.

We drew 1000 datasets with sample sizes shown in Table 1. These sample sizes give us 80% power to detect the desired effect for each LR model. A generative model was trained for each real data sample using sequential synthesis and CTGAN, which gave us 2000 generative models for each dataset. For each generative model 20 synthetic datasets were generated and these were used in our analysis. LR models using *m* = 1 … 20 datasets were fitted, and their results combined, and the eight metrics described above computed for each *m*, as well as membership disclosure. When generating synthetic datasets, we also evaluated the impact of data amplification. We evaluated four levels of amplification: 1×, 2×, 5×, and 10×. The baseline for the amplification is the 80% power sample size in Table 1 (i.e., amplification is equal to $k/n$).

The failure rate during the simulations was highest with the DCCG dataset using the sequential synthesis method at 1.29% of the 1000 simulation runs. This could be due to the failure of the generative model or lack of convergence in the LR model. The failure rate for the sequential generative model with the N0147 was 0.03%, and for CTGAN with the N0147 dataset was 0.16%. For the other dataset—generative model combinations there were not failures. When failures occurred, they were treated as missing observations in the analysis.

#### Statistical testing

We do not perform statistical significance tests to compare the different metrics because in the context of a simulation these are not informative. The number of simulation runs can be increased to make very small effects statistically significant. Therefore, the results are presented descriptively which gives us the information we need to evaluate replicability and privacy.

### Neutrality of simulation study

This simulation was intended to be a neutral comparison study so as not to favor any particular generative model^99^. We argue that we meet two of the criteria for a neutral comparative study completely and meet the third one partially. First, the purpose of our study was to evaluate the replicability across common generative models rather than to evaluate a new proposed generative model. Both of the generative models included in our study have been used often in research and practice. Second, the evaluation criteria were selected based on the existing literature and we have tried to be more inclusive with respect to the selection of metrics. Therefore, there was a rational process to the choice of metrics. For the third criterion, while we are neutral with respect to the two methods included in our study in that we have evaluated them both before^25,94,95^, we have also performed more research and applied work with the sequential synthesis method^9,28^.

### Ethics

The protocol for this study was approved by the Veritas IRB protocol number 2021-2882-7683-1, and the Children’s Hospital of Eastern Ontario Research Institute research ethics board protocol number 23/23X. The use of the DCCG dataset was approved by the Danish Data Protection Agency (Datatilsynet) number RN-2018-94. This study was performed in accordance with relevant guidelines and regulations. All the datasets used were provided to the research team for secondary analysis and they were already deemed to be de-identified/anonymized. Therefore, the Children’s Hospital of Eastern Ontario Research Institute Research Ethics Board did not require additional consent from the data subjects for this study.

## Results

We present the results for the N0147 dataset in the main body of the paper and summarize the findings for the other two datasets which are included in the supplementary materials. We generally found that the CTGAN replicability results were quite poor, and we include those results in the supplementary materials.

In the results we will refer to the findings for a single dataset without the application of the combining rules as “single”. When the parameter estimates and their CI values are adjusted using the combining rules in “Adjustment using multiple imputation combining rules” we will refer to the results as “multiple”. Even for $m = 1$, when the combining rules are applied the adjusted variance is $T_{f} = 2\overline{v}_{m}$ with no amplification. This is different from the “single” variance ($v_{1}$) which would be just the computed value from the fitted model. Therefore, in the “multiple” case, even for one synthetic dataset, the parameter variance is adjusted upward to account for the generative process.

**Figure 3:**
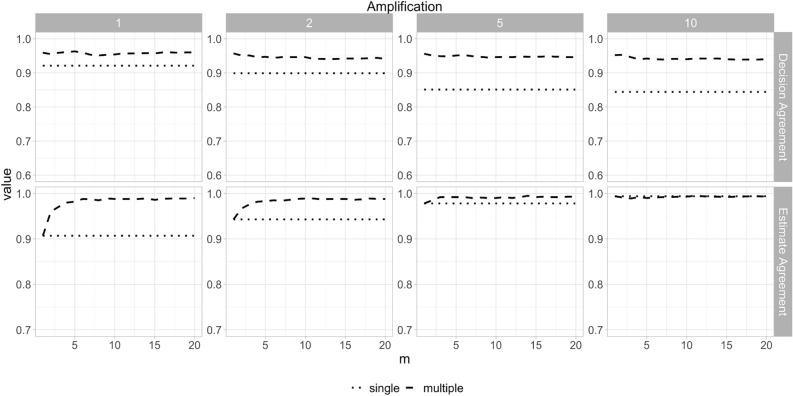
Decision and estimate agreement for the N0147 colon cancer dataset using the sequential synthesis method. The amplification value indicates the multiple of the sample size shown in Table 1 (1420).

**Figure 4:**
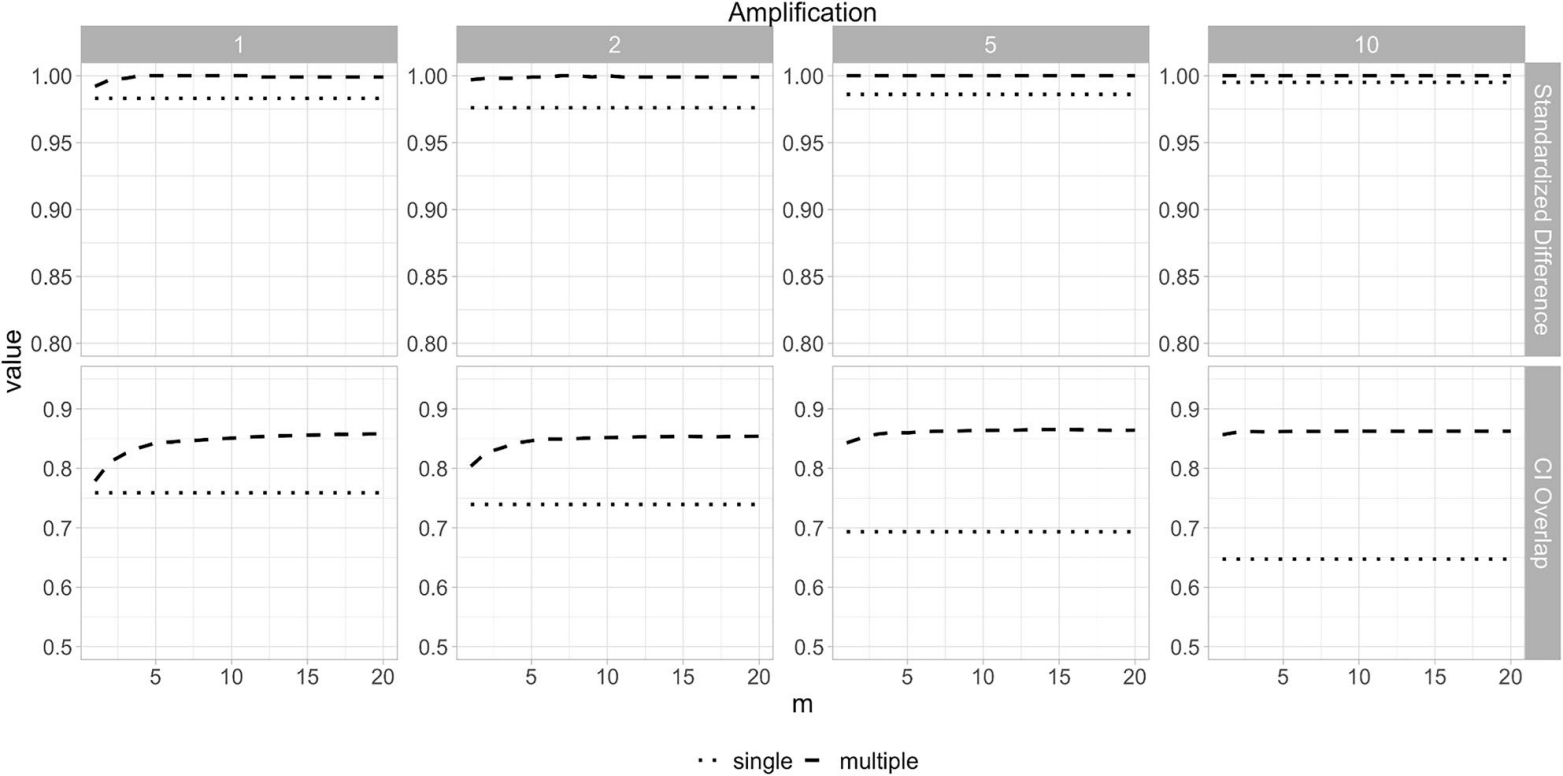
Standardized difference and confidence interval overlap for the N0147 colon cancer dataset using the sequential synthesis method. The amplification value indicates the multiple of the sample size shown in Table 1 (1420).

For multiple dataset results, the decision agreement results for the N0147 dataset are shown in Fig. 3, and are high (all above the 0.8 threshold) for all values of $m$, and decrease slightly as $m$ increases. The estimate agreement reaches a plateau at $m = 5$ and at that plateau is also above the 0.83 threshold. Standardized difference is shown in Fig. 4 along with CI overlap. Standardized difference is consistently above the 0.95 value, and the CI overlap results are also quite high (mostly above 0.8) and increase with higher values of $m$, reaching a plateau at $m = 5$. These observations are consistent with the DCCG and CCHS datasets shown in the supplementary materials.

Data amplification affects the single dataset results for estimate agreement, and this is consistent with the pattern that for larger synthetic datasets the parameter estimate will converge to the true mean. CI overlap deteriorated for the single results with amplification. Decision agreement is not affected by amplification since the results were statistically significant and therefore narrower confidence intervals did not change that conclusion. Amplification did not have a material impact on the “multiple” results.

The N0147 and DCCG results using CTGAN provided in the supplementary materials are comparable to Figs. 3 and 4 with higher agreement, standardized difference, and CI overlap with higher values of $m$, reaching an acceptable plateau at $m = 5$. The CCHS results with CTGAN are quite poor, with low estimate agreement and confidence interval overlap results.

**Figure 5:**
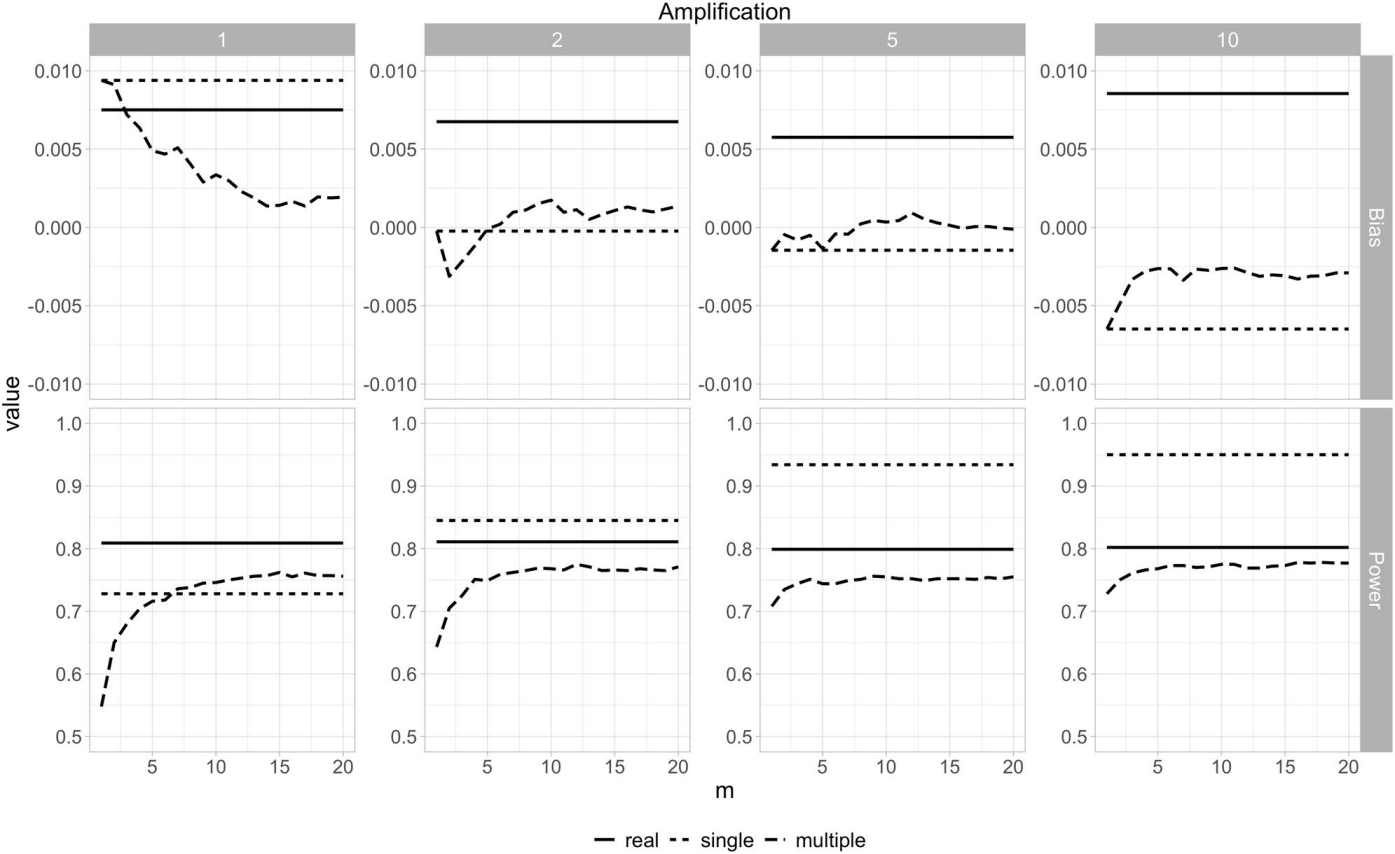
The bias and power for the N0147 colon cancer dataset using the sequential synthesis method. The amplification value indicates the multiple of the sample size shown in Table 1 (1420).

The bias and power results across different values of *m* for the N0147 dataset are shown in Fig. 5 at different levels of amplification. The bias is consistently close to zero, and power is close to the nominal 80% level. Bias and power tend to plateau with higher values of $m = 10$. Amplification does help increase power only slightly for the “multiple” datasets. As expected, “single” power increases with amplification because it is a simple increase in sample size without adjustment of the variance.

**Figure 6:**
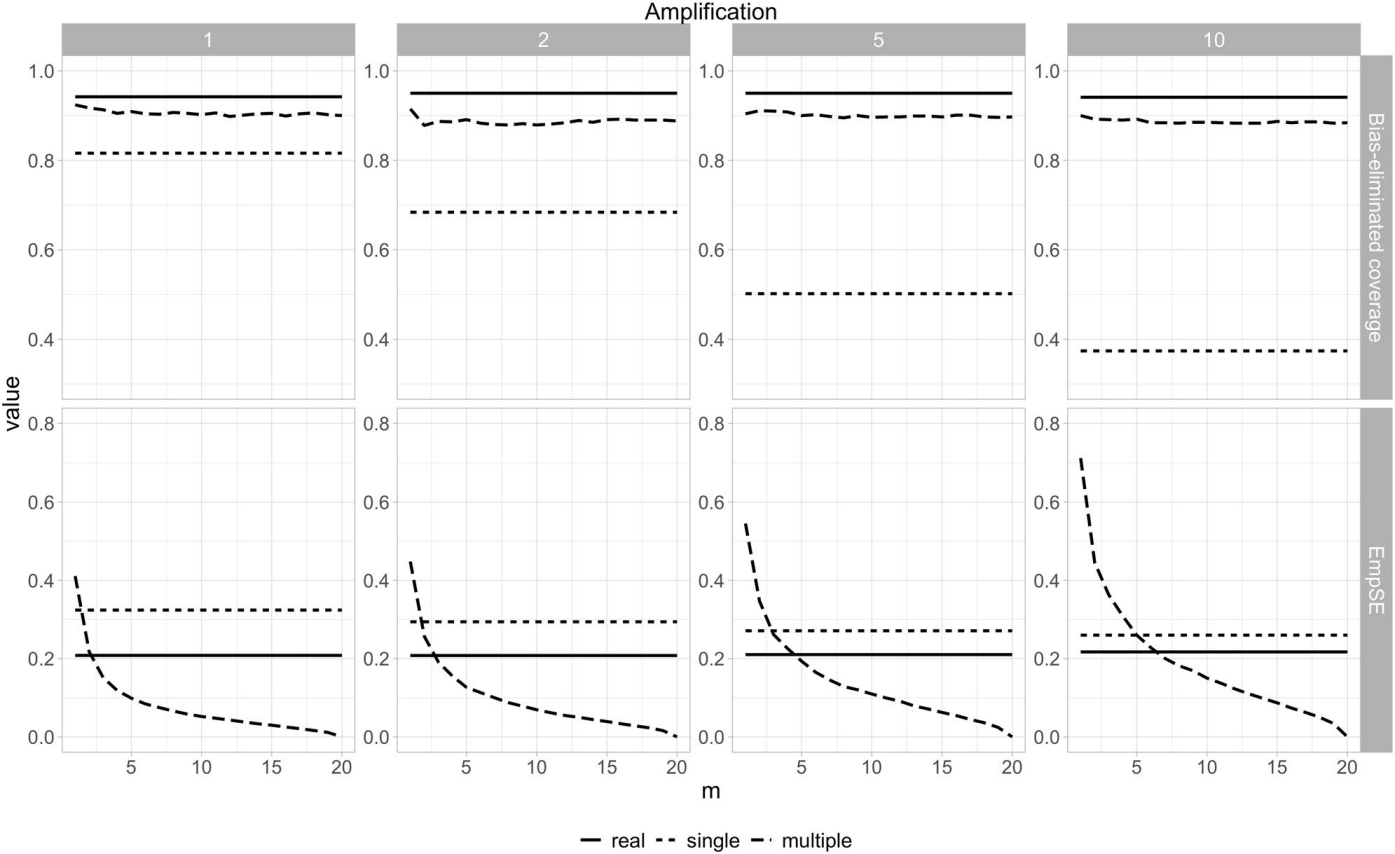
The coverage and empirical SE for the N0147 colon cancer dataset using the sequential synthesis method. The amplification value indicates the multiple of the sample size shown in Table 1 (1420).

The bias eliminated coverage and empirical SE plots across different values of *m* for the N0147 dataset are shown in Fig. 6 at different levels of amplification. The coverage of the adjusted parameters is consistently close to the 95% nominal level. Empirical SE decreases towards zero and plateaus at higher values of *m*. This is not surprising since as $m$ increases the average variance values move closer to the average across simulation runs—the combined estimates become more consistent. Amplification does not change the general patterns observed. The coverage and empirical SE for the “single” results tend to be poor, with coverage far from the nominal 95% level, and empirical SE being quite high.

The results for the CCHS and DCCG datasets generated by sequential synthesis are very similar to the N0147 ones for the population inference results. These results are included in the supplementary materials.

For CTGAN the findings, included in the supplementary materials, are different. Bias is high and power is quite poor for the N0147 and CCHS datasets. But CTGAN performs quite well on these metrics for the DCCG dataset. Similarly, coverage for N0147 and CCHS exceeds the nominal level, but is at the nominal level for the DCCG dataset. Empirical SE performs similarly across all datasets with a gradual convergence to zero as more replicates are generated. Amplification did not change these patterns for the adjusted datasets.

The membership disclosure results are shown in Table 4. The value does not vary by the number of synthetic datasets that are generated. This is because the risk reaches its maximum with one dataset, and the values in Table 4 reflect their average. The risk value is low (below the suggested 0.2 threshold in the literature) suggesting that the privacy risks are acceptable for the synthetic data irrespective of the number of data replicates. The conclusions are similar for the CTGAN membership disclosure, also shown in the supplementary materials.

**Table 4:** Averaged membership disclosure values for the three datasets using the sequential synthesis generative model.

	Membership disclosure
N0147	0.0035
DCCG	0.08045
CCHS	0.00547

Overall, for sequential synthesis, the “multiple” results are superior than the “single” results. In many cases the evaluative metrics plateau at approximately $m = 10$. This is the case across all eight criteria that are used. For the privacy criterion there is no difference between “single” and “multiple” results.

Note that the Monte Carlo standard error^31^ was also computed for the various evaluative metrics. This was negligibly low and would not be visible in the plots.

## Discussion

### Summary

In this study we evaluated the replicability of findings using fully synthetic datasets through a series of simulations. Two sets of evaluative criteria were used to assess replicability: (a) the similarity of analysis findings to those from real data, and (b) the validity of population inferences. The simulations were based on three heterogeneous datasets covering multiple diseases, conditions, data collection modalities and jurisdictions. Two different, but commonly used, types of generative models were evaluated: a sequential synthesis approach using decision trees and a conditional generative adversarial network approach. The assumed analytical workload was logistic regression.

Generating multiple datasets using sequential synthesis and combining the parameter estimates provides for better replication of results than using a single synthetic dataset without any adjustments to the estimates and confidence intervals.

The results allow us to respond to the questions we posed at the outset of the study:

**Table:** 

Q1	Applying combining rules from 10 synthetic datasets was sufficient to ensure good performance across all of our eight metrics for data generated using sequential synthesis. The replicability of results of single synthetic datasets without the use of any combining rule adjustments was generally poor, and can be misleading
Q2	Membership disclosure risk is consistently below the threshold across all generative models and is not materially affected by the value of $m$ or by amplification
Q3	The generation of amplified datasets only had a marginal impact on replicability in general, and more importantly had a very marginal effect on statistical power when the combining rules were applied
Q4	The replicability of analyses when the synthetic data was generated using sequential synthesis was high, but for CTGAN replicability was quite poor in some datasets, with decision and estimate agreement severely impacted, as well as power being far off the nominal value and high degrees of bias on some datasets. Therefore, the ability to replicate real data results from synthetic data will depend on the type of generative model being used

Our results indicate that sequential synthesis gave better replicability than CTGAN. These results are consistent with previous comparative evidence on oncology data whereby a sequential synthesis generative model utilizing decision trees had better utility than a GAN^46,94^. There are also implementation differences that may contribute to sequential synthesis performing better. Our sequential synthesis implementation had a more complete process for handling missing data compared to the open source Synthetic Data Vault (SDV) implementation of CTGAN that we used^90,100^. We observed that SDV generative models were not able to reproduce the missingness patterns in the synthetic data as well. Furthermore, the SDV implementation had limited hyperparameter tuning.

While GANs have been used extensively for SDG^80,81^, there is evidence that performance can vary significantly across different GAN architectures^101^. This dependence on the type of generative model, even within the same class, suggests that the kind of evaluation we presented here should be conducted for each type of generative model when applied in practice.

### Application in practice

Our results indicate that generating a single dataset and performing analysis on that without any adjustments to model parameters and standard errors can result in low replicability. Analytic conclusions should be drawn from models fitted on ten synthetic datasets and their parameters combined to ensure replicability of analyses.

This does not necessarily mean that generative models should be provided to data users to allow them to generate multiple datasets themselves. In general, machine learning (ML) models are known to be susceptible to adversarial attacks that can reveal sensitive information about the individuals in the training datasets^102,103^. Therefore, it has been argued that sharing ML models may lead to different types of disclosure risks, making (unprotected) ML models equivalent to personally identifiable information^104^. Hence, there may be additional privacy risk from sharing generative models. Instead, we propose that data custodians should share ten instances of synthetic datasets rather than single synthetic datasets to ensure the replicability of findings.

There is equivocal value to amplification of synthetic data for statistical analyses. Because of the relatively low computational burden of amplification, a 5× (or even 10×) amplification for the ten generated datasets can marginally improve replicability, although one can make the counterargument that the additional complexity of handling larger datasets would not provide a meaningful return in terms of replicability.

Our methodology can also serve as a general framework for evaluating and comparing the replicability of synthetic datasets. Replicability is only one dimension of the utility of synthetic datasets and generative models, but an important one.

### Limitations

Our analytic workload was logistic regression models. This type of analysis is one of the most common in health research and therefore the results should still have broad applicability^62^. However, other types statistical models should be evaluated in future work.

Our study was focused on evaluating the replicability using fully synthetic datasets. We did not consider partially synthetic datasets nor hybrid synthetic datasets, which may produce a different set of recommendations. We also did not consider other utility metrics, such as the fidelity of the synthetic data. Arguably, fidelity is mostly relevant if it enables replicability^25^, and therefore having a framework for assessing replicability is a necessary condition for assessing utility in general.

We did not examine the impact of generating multiple synthetic datasets on the results of machine learning models and prognostic model prediction accuracy on unseen cases. We limited our investigations to the commonly used logistic regression models and parameter inferences only.

Our results are limited by the characteristics of the datasets that were used. While there was heterogeneity in these datasets in terms of type, jurisdiction, and context, additional evaluations using our replicability framework would be of value.

### Supplementary Information

Supplementary Information.

## Data Availability

The materials from this work have been deposited in OSF at (10.17605/OSF.IO/VSKU2). The N0147 dataset can be requested from Project Data Sphere. Access to the master CCHS dataset can be requested from Statistics Canada, however, a public use file from Statistics Canada for this dataset has been deposited in the OSF repository for this project to enable reproducibility. The DCCG dataset can be requested from the Danish Colon Cancer Registry. The Python and R code used in the analysis and visualizations have been deposited in the OSF repository, as well as the interim results used for the plots of the results. The original protocol and one amendment that were submitted to the research ethics board for this study are also included in the OSF repository. These protocols follow the format required by the research ethics board at the CHEO Research Institute that reviewed the study.

## References

[1] Foraker RE, Yu SC, Gupta A (2020). Spot the difference: Comparing results of analyses from real patient data and synthetic derivatives. JAMIA Open.

[2] Tucker A, Wang Z, Rotalinti Y (2020). Generating high-fidelity synthetic patient data for assessing machine learning healthcare software. npj Digit. Med..

[3] Wang, Z., Myles, P. & Tucker, A. Generating and evaluating synthetic UK primary care data: Preserving data utility patient privacy. In *2019 IEEE 32nd International Symposium on Computer-Based Medical Systems (CBMS)*, Cordoba. 126–31. 10.1109/CBMS.2019.00036 (2019).

[4] Wang Z, Myles P, Tucker A (2021). Generating and evaluating cross-sectional synthetic electronic healthcare data: Preserving data utility and patient privacy. Comput. Intell..

[5] Reiner Benaim A, Almog R, Gorelik Y (2020). Analyzing medical research results based on synthetic data and their relation to real data results: Systematic comparison from five observational studies. JMIR Med. Inform..

[6] Mendelevitch, O. & Lesh, M.D. *Fidelity and Privacy of Synthetic Medical Data*. arXiv:210108658 [cs] (2021).

[7] Muniz-Terrera G, Mendelevitch O, Barnes R (2021). Virtual cohorts and synthetic data in dementia: An illustration of their potential to advance research. Front. Artif. Intell..

[8] Foraker R, Guo A, Thomas J (2021). Analyses of original and computationally-derived electronic health record data: The National COVID Cohort Collaborative. J. Med. Internet Res..

[9] Azizi Z, Zheng M, Mosquera L (2021). Can synthetic data be a proxy for real clinical trial data ? A validation study. BMJ Open.

[10] El Emam K, Mosquera L, Jonker E (2021). Evaluating the utility of synthetic COVID-19 case data. JAMIA Open..

[11] Beaulieu-Jones BK, Wu ZS, Williams C (2019). Privacy-preserving generative deep neural networks support clinical data sharing. Circ. Cardiovasc. Qual. Outcomes.

[12] Polonetsky, J. & Renieris, E. *10 Privacy Risks and 10 Privacy Technologies to Watch in the Next Decade. Future of Privacy Forum* (2020).

[13] Guo A, Foraker RE, MacGregor RM (2020). The use of synthetic electronic health record data and deep learning to improve timing of high-risk heart failure surgical intervention by predicting proximity to catastrophic decompensation. Front. Digit. Health.

[14] Haendel MA, Chute CG, Bennett TD (2021). The National COVID Cohort Collaborative (N3C): Rationale, design, infrastructure, and deployment. J. Am. Med. Inform. Assoc..

[15] CMS. *CMS 2008–2010 Data Entrepreneurs’ Synthetic Public Use File* (*DE-SynPUF*). https://www.cms.gov/Research-Statistics-Data-and-Systems/Downloadable-Public-Use-Files/SynPUFs/DE_Syn_PUF. Accessed 17 July 2022 (2022).

[16] *Generating and Evaluating Synthetic UK Primary Care Data: Preserving Data Utility & Patient Privacy-IEEE Conference Publication*. https://ieeexplore-ieee-org.proxy.bib.uottawa.ca/abstract/document/8787436. Accessed 31 Aug 2019 (2019).

[17] Synthetic data at CPRD. *Medicines & Healthcare products Regulatory Agency*. https://www.cprd.com/content/synthetic-data. Accessed 24 Sep 2020 (2020).

[18] NHS England. *A&E Synthetic Data*. https://data.england.nhs.uk/dataset/a-e-synthetic-data. Accessed 16 July 2022 (2022)

[19] Synthetic dataset. *Integraal Kankercentrum Nederland*. https://iknl.nl/en/ncr/synthetic-dataset . Accessed 20 Nov 2021 (2021).

[20] The Simulacrum. *The Simulacrum*. https://simulacrum.healthdatainsight.org.uk/ . Accessed 27 Nov 2021 (2021).

[21] SNDS synthétiques. *Systeme National des Donnees de Sante*. https://documentation-snds.health-data-hub.fr/formation_snds/donnees_synthetiques/. Accessed 20 Jan 2022 (2021).

[22] #opendata4covid19 Website User Manual. https://rtrod-assets.s3.ap-northeast-2.amazonaws.com/static/tools/manual/COVID-19+website+manual_v2.1.pdf . Accessed 8 Apr 2020 (2020).

[23] Lun R, Siegal D, Ramsay T (2024). Synthetic data in cancer and cerebrovascular disease research: A novel approach to big data. PLOS ONE..

[24] Karr A, Koonen C, Oganian A (2006). A framework for evaluating the utility of data altered to protect confidentiality: The American Statistician: Vol. 60, No. 3. Am. Stat..

[25] Emam KE, Mosquera L, Fang X (2022). Utility metrics for evaluating synthetic health data generation methods: Validation study. JMIR Med. Inform..

[26] Goncalves A, Ray P, Soper Bx (2020). Generation and evaluation of synthetic patient data. BMC Med. Res. Methodol..

[27] Platzer, M. & Reutterer, T. *Holdout-Based Fidelity and Privacy Assessment of Mixed-Type Synthetic Data*. arXiv:210400635 [cs, stat] (2021). 10.3389/fdata.2021.679939 PMC8276128 34268491

[28] El Emam K, Mosquera L, Zheng C (2020). Optimizing the synthesis of clinical trial data using sequential trees. J. Am. Med. Inform. Assoc..

[29] National Academies of Sciences, Engineering, and Medicine. *Reproducibility and Replicability in Science*. http://www.ncbi.nlm.nih.gov/books/NBK547537/. Accessed 28 July 2023 (National Academies Press (US), 2019). 31596559

[30] Grund, S., Lüdtke, O. & Robitzsch, A. Using synthetic data to improve the reproducibility of statistical results in psychological research. *Psychol. Methods* (2022). 10.1037/met0000526 35925728

[31] Morris TP, White IR, Crowther MJ (2019). Using simulation studies to evaluate statistical methods. Stat. Med..

[32] Rubin D (1993). Discussion: Statistical disclosure limitation. J. Off. Stat..

[33] Raghunathan T, Reiter J, Rubin D (2003). Multiple imputation for statistical disclosure control. J. Off. Stat..

[34] Reiter JP (2002). Satisfying disclosure restrictions with synthetic data sets. J. Off. Stat..

[35] Raab GM, Nowok B, Dibben C (2016). Practical data synthesis for large samples. J. Priv. Confident..

[36] Reiter JP (2004). New approaches to data dissemination: A glimpse into the future (?). Chance.

[37] Park N, Mohammadi M, Gorde K (2018). Data synthesis based on generative adversarial networks. Proc. VLDB Endow..

[38] Hu, J. *Bayesian Estimation of Attribute and Identification Disclosure Risks in Synthetic Data*. arXiv:180402784 [stat] (2018).

[39] Taub J, Elliot M, Pampaka M, Domingo-Ferrer J, Montes F (2018). Differential correct attribution probability for synthetic data: An exploration. Privacy in Statistical Databases.

[40] Hu J, Reiter JP, Wang Q, Domingo-Ferrer J (2014). Disclosure risk evaluation for fully synthetic categorical data. Privacy in Statistical Databases.

[41] Wei L, Reiter JP (2016). Releasing synthetic magnitude microdata constrained to fixed marginal totals. Stat. J. IAOS.

[42] Ruiz N, Muralidhar K, Domingo-Ferrer J, Domingo-Ferrer J, Montes F (2018). On the privacy guarantees of synthetic data: A reassessment from the maximum-knowledge attacker perspective. Privacy in Statistical Databases.

[43] Reiter JP (2005). Releasing multiply imputed, synthetic public use microdata: An illustration and empirical study. J. R. Stat. Soc. Ser. A (Statistics in Society).

[44] Zhang Z, Yan C, Mesa DA (2021). Ensuring electronic medical record simulation through better training, modeling, and evaluation. J. Am. Med. Inform. Assoc..

[45] Zhang Z, Yan C, Lasko TA (2020). SynTEG: A framework for temporal structured electronic health data simulation. J. Am. Med. Inform. Assoc..

[46] Goncalves A, Ray P, Soper B (2020). Generation and evaluation of synthetic patient data. BMC Med. Res. Methodol..

[47] Hilprecht B, Härterich M, Bernau D (2019). Monte Carlo and reconstruction membership inference attacks against generative models. Proc. Priv. Enhanc. Technol..

[48] Taub J, Elliot M, Sakshaug W (2020). The impact of synthetic data generation on data utility with application to the 1991 UK samples of anonymised records. Trans Data Priv..

[49] Drechsler J, Dundler A, Bender S (2008). A new approach for disclosure control in the IAB establishment panel—Multiple imputation for a better data access. AStA Adv. Stat. Anal..

[50] Loong B, Rubin DB (2017). Multiply-imputed synthetic data: Advice to the imputer. J. Off. Stat..

[51] Loong B, Zaslavsky AM, He Y (2013). Disclosure control using partially synthetic data for large-scale health surveys, with applications to CanCORS. Stat. Med..

[52] Reiter J (2003). Inference for partially synthetic, public use microdata sets. Surv. Methodol..

[53] van der Ploeg T, Austin PC, Steyerberg EW (2014). Modern modelling techniques are data hungry: A simulation study for predicting dichotomous endpoints. BMC Med. Res. Methodol..

[54] CEO Life Sciences Consortium. Share, Integrate & Analyze Cancer Research Data. *Project Data Sphere*. https://projectdatasphere.org/projectdatasphere/html/home. Accessed 11 July 2019 (2019).

[55] Alberts SR, Sargent DJ, Nair S (2012). Effect of oxaliplatin, fluorouracil, and leucovorin with or without cetuximab on survival among patients with resected stage III colon cancer: A randomized trial. JAMA.

[56] El-Hussuna A, Lytras T, Bruun NH (2022). Extended right-sided colon resection does not reduce the risk of colon cancer local-regional recurrence: Nationwide population-based study from Danish Colorectal Cancer Group Database. Dis. Colon Rectum.

[57] Chen H, Cohen P, Chen S (2010). How big is a big odds ratio? Interpreting the magnitudes of odds ratios in epidemiological studies. Commun. Stat.-Simul. Comput..

[58] Schäfer T, Schwarz MA (2019). The meaningfulness of effect sizes in psychological research: Differences between sub-disciplines and the impact of potential biases. Front. Psychol..

[59] Song F, Parekh S, Hooper L (2010). Dissemination and publication of research findings : An updated review of related biases. Health Technol. Assess..

[60] Demidenko E (2007). Sample size determination for logistic regression revisited. Stat. Med..

[61] Hsieh FY, Bloch DA, Larsen MD (1998). A simple method of sample size calculation for linear and logistic regression. Stat. Med..

[62] Collins GS, Reitsma JB, Altman DG (2015). Transparent reporting of a multivariable prediction model for individual prognosis or diagnosis (TRIPOD): The TRIPOD statement. BMJ.

[63] Christodoulou E, Ma J, Collins GS (2019). A systematic review shows no performance benefit of machine learning over logistic regression for clinical prediction models. J. Clin. Epidemiol..

[64] Dankar FK, Ibrahim M (2021). Fake it till you make it: Guidelines for effective synthetic data generation. Appl. Sci..

[65] Dahdaleh FS, Sherman SK, Poli EC (2018). Obstruction predicts worse long-term outcomes in stage III colon cancer: A secondary analysis of the N0147 trial. Surgery.

[66] Maclagan LC, Park J, Sanmartin C (2014). The CANHEART health index: A tool for monitoring the cardiovascular health of the Canadian population. CMAJ.

[67] Azizi Z, Lindner S, Shiba Y (2023). A comparison of synthetic data generation and federated analysis for enabling international evaluations of cardiovascular health. Sci. Rep..

[68] European Society of Coloproctology Collaborating Group (2020). Predictors for anastomotic leak, postoperative complications, and mortality after right colectomy for cancer: Results from an International Snapshot Audit. Dis. Colon Rectum.

[69] 2017 and 2015 European Society of Coloproctology (ESCP) collaborating groups. The impact of conversion on the risk of major complication following laparoscopic colonic surgery: An international, multicentre prospective audit. *Colorectal Dis*. **20** (Suppl 6), 69–89 (2018). 10.1111/codi.14371 30255643

[70] Reiter J (2005). Using CART to generate partially synthetic, public use microdata. J. Off. Stat..

[71] Drechsler J, Reiter JP (2011). An empirical evaluation of easily implemented, nonparametric methods for generating synthetic datasets. Comput. Stat. Data Anal..

[72] Arslan RC, Schilling KM, Gerlach TM (2021). Using 26,000 diary entries to show ovulatory changes in sexual desire and behavior. J. Pers. Soc. Psychol..

[73] Bonnéry D, Feng Y, Henneberger AK (2019). The promise and limitations of synthetic data as a strategy to expand access to state-level multi-agency longitudinal data. J. Res. Educ. Effect..

[74] Sabay A, Harris L, Bejugama V (2018). Overcoming small data limitations in heart disease prediction by using surrogate data. SMU Data Sci. Rev..

[75] Freiman, M., Lauger, A. & Reiter, J. *Data Synthesis and Perturbation for the American Community Survey at the U.S. Census Bureau*. US Census Bureau. https://www.census.gov/library/working-papers/2018/adrm/formal-privacy-synthetic-data-acs.html. Accessed 24 Feb 2020 (2017).

[76] Nowok, B. *Utility of Synthetic Microdata Generated Using Tree-Based Methods*. https://unece.org/statistics/events/SDC2015 (Helsinki, 2015).

[77] Nowok B, Raab GM, Dibben C (2017). Providing bespoke synthetic data for the UK longitudinal studies and other sensitive data with the synthpop package for R 1. Stat. J. IAOS.

[78] Quintana DS (2020). A synthetic dataset primer for the biobehavioural sciences to promote reproducibility and hypothesis generation. eLife.

[79] Little, C., Elliot, M., Allmendinger, R. *et al.**Generative Adversarial Networks for Synthetic Data Generation: A Comparative Study*. Vol. 17. https://unece.org/statistics/documents/2021/12/working-documents/generative-adversarial-networks-synthetic-data. (United Nations Economic Commission for Europe, 2021).

[80] Hernandez M, Epelde G, Alberdi A (2022). Synthetic data generation for tabular health records: A systematic review. Neurocomputing..

[81] Jacobs F, D’Amico S, Benvenuti C (2023). Opportunities and challenges of synthetic data generation in oncology. JCO Clin. Cancer Inform..

[82] Ghosheh GO, Li J, Zhu T (2024). A survey of generative adversarial networks for synthesizing structured electronic health records. ACM Comput. Surv..

[83] Chin-Cheong, K., Sutter, T. & Vogt, J.E. *Generation of Heterogeneous Synthetic Electronic Health Records using GANs*. 10.3929/ethz-b-000392473 (2019).

[84] Choi, E., Biswal, S., Malin, B. *et al.**Generating Multi-Label Discrete Patient Records Using Generative Adversarial Networks*. arXiv:170306490 [cs] (2017).

[85] Yan, C., Zhang, Z., Nyemba, S. *et al.**Generating Electronic Health Records with Multiple Data Types and Constraints*. arXiv:200307904 [cs, stat] (2020). PMC8075510 33936510

[86] Bühlmann P, Hothorn T (2007). Boosting algorithms: Regularization. Predict. Model Fit. Stat. Sci..

[87] Ke, G., Meng, Q., Finley, T. *et al.* LightGBM: A highly efficient gradient boosting decision tree. In *Advances in Neural Information Processing Systems* (Guyon, I., Luxburg, U.V., Bengio, S. *et al.* eds.). Vol. 30. 3146–3154. http://papers.nips.cc/paper/6907-lightgbm-a-highly-efficient-gradient-boosting-decision-tree.pdf. Accessed 15 Oct 2020 (Curran Associates, Inc., 2017).

[88] Snoek, J., Larochelle, H. & Adams, R.P. Practical Bayesian optimization of machine learning algorithms. In *Proceedings of the 25th International Conference on Neural Information Processing Systems*. Vol. 2. 2951–2959. https://papers.nips.cc/paper_files/paper/2012/hash/05311655a15b75fab86956663e1819cd-Abstract.html (Curran Associates Inc., 2012).

[89] Jones MC (1993). Simple boundary correction for kernel density estimation. Stat. Comput..

[90] Xu, L., Skoularidou, M., Cuesta-Infante, A. *et al.* Modeling tabular data using conditional GAN. In *Advances in Neural Information Processing Systems *(Wallach, H., Larochelle, H., d’Alche-Buc, F. *et al.* eds.). 7335–7345. https://papers.nips.cc/paper/2019/hash/254ed7d2de3b23ab10936522dd547b78-Abstract.html. Accessed 2 Oct 2021 (Curran Associates, Inc., 2019).

[91] Bourou S, El Saer A, Velivasaki T (2021). A review of tabular data synthesis using GANs on an IDS dataset. Information.

[92] Mirza, M. & Osindero, S. *Conditional Generative Adversarial Nets*. 10.48550/arXiv.1411.1784 (2014).

[93] Xu, L., Skoularidou, M., Cuesta-Infante, A. *et al.* Modeling tabular data using conditional GAN. In *Advances in Neural Information Processing Systems*. https://papers.nips.cc/paper/2019/hash/254ed7d2de3b23ab10936522dd547b78-Abstract.html (2019).

[94] El Kababji, S., Mitsakakis, N., Fang, X. *et al.* Evaluating the utility and privacy of synthetic breast cancer clinical trial datasets. *JCO CCI***(accepted)**. 10.1200/CCI.23.00116 PMC10703127 38011617

[95] El Emam K, Mosquera L, Fang X (2022). Validating a membership disclosure metric for synthetic health data. JAMIA Open..

[96] Cancer of the Colon and Rectum-Cancer Stat Facts. *SEER*. https://seer.cancer.gov/statfacts/html/colorect.html. Accessed 9 Oct 2021 (2021).

[97] Iversen LH, Green A, Ingeholm P (2016). Improved survival of colorectal cancer in Denmark during 2001–2012—The efforts of several national initiatives. Acta Oncol..

[98] Burton A, Altman DG, Royston P (2006). The design of simulation studies in medical statistics. Stat. Med..

[99] Boulesteix A-L, Lauer S, Eugster MJA (2013). A plea for neutral comparison studies in computational sciences. PLOS ONE.

[100] Patki, N., Wedge, R. & Veeramachaneni, K. The synthetic data vault. In *2016 IEEE International Conference on Data Science and Advanced Analytics (DSAA)*. 399–410. 10.1109/DSAA.2016.49 (IEEE, 2016).

[101] Yan, C., Yan, Y., Wan, Z. *et al.**A Multifaceted Benchmarking of Synthetic Electronic Health Record Generation Models*. 10.48550/arXiv.2208.01230 (2022). 10.1038/s41467-022-35295-1 PMC9734113 36494374

[102] De Cristofaro E (2021). A critical overview of privacy in machine learning. IEEE Secur. Privacy.

[103] Shafee A, Awaad TA (2021). Privacy attacks against deep learning models and their countermeasures. J. Syst. Architect..

[104] Veale M, Binns R, Edwards L (2018). Algorithms that remember: Model inversion attacks and data protection law. Philos. Trans. R. Soc. A Math. Phys. Eng. Sci..

[105] Klein RA, Ratliff KA, Vianello M (2014). Investigating variation in replicability: A “many labs” replication project. Soc. Psychol..

[106] Camerer CF, Dreber A, Holzmeister F (2018). Evaluating the replicability of social science experiments in nature and science between 2010 and 2015. Nat. Hum. Behav..

[107] Open Science Collaboration. Estimating the reproducibility of psychological science. *Science* **349**, aac4716 (2015). 10.1126/science.aac4716 26315443

[108] Franklin JM, Pawar A, Martin D (2020). Nonrandomized real-world evidence to support regulatory decision making: Process for a randomized trial replication project. Clin. Pharmacol. Ther..

[109] Crown W, Dahabreh IJ, Li X (2023). Can observational analyses of routinely collected data emulate randomized trials? Design and feasibility of the observational patient evidence for regulatory approval science and understanding disease project. Value Health..

[110] Yoon D, Jeong HE, Park S (2023). Real-world data emulating randomized controlled trials of non-vitamin K antagonist oral anticoagulants in patients with venous thromboembolism. BMC Med..

[111] Wang SV, Schneeweiss S, RCT-DUPLICATE Initiative (2023). Emulation of randomized clinical trials with nonrandomized database analyses: Results of 32 clinical trials. JAMA.

[112] Franklin JM, Patorno E, Desai RJ (2021). Emulating randomized clinical trials with nonrandomized real-world evidence studies. Circulation..

[113] Patil P, Peng RD, Leek JT (2016). What should researchers expect when they replicate studies? A statistical view of replicability in psychological science. Perspect. Psychol. Sci..

