# Supplementary material for: An evaluation of the replicability of analyses using synthetic health data
Source: Sci Rep. 2024 Mar 24;14:6978. doi: 10.1038/s41598-024-57207-7 (PMC10960851; doi:10.1038/s41598-024-57207-7)
Supplement: Supplementary file 1 — Supplementary Information. [file 41598_2024_57207_MOESM1_ESM.pdf]

Supplementary Materials

1. Description of Datasets

This section provides a summary of each dataset.

1.1 N0147

Trial N0147 was a randomized trial of 2,686 patients with stage 3 colon adenocarcinoma that were randomly assigned to adjuvant regimens with or without Cetuximab. After resection of colon cancer, Cetuximab was added to the modified sixth version of the FOLFOX regimen including oxaliplatin plus 5-fluorouracil and leucovorin (mFOLFOX6), fluorouracil, leucovorin, and irinotecan (FOLFIRI), or a hybrid regimen consisting of mFOLFOX6 followed up by FOLFIRI [1]. Our focus is on the secondary retrospective analysis of N0147 (the published secondary analysis) [2].

The primary endpoint in the original trial was disease-free survival (DFS), defined as time from random allocation to the first of either tumor recurrence or death from any cause. Secondary trial endpoints were time to recurrence (TTR) and overall survival (OS). TTR was measured from random allocation to tumor recurrence, whereas OS was from random allocation to death from any cause. OS was censored at 8 years, whereas DFS and TTR were censored at 5 years. Patients who died without recurrence were censored for TTR at the time of death. Patients who were lost to follow-up were censored at the date of their most recent disease assessment or contact.

Participants in the control "chemotherapy-only" arm (FOLFOX, FOLFIRI or hybrid regimen without Cetuximab) were analyzed in the published secondary analysis, which consisted of 1,543 patients. Presentation with acute obstruction of the bowel is a known risk factor for poor prognosis in patients with colon cancer [3], [4]. The main objective of this secondary analysis was to assess the role of obstruction presentation as an independent risk factor for predicting outcomes in patients with stage III colon cancer. The primary endpoint of the in the published secondary analysis was disease free survival (DFS), and the secondary endpoint was overall survival (OS), and both DFS and OS were censored at five years.

The covariates in the published secondary analysis comprised of three types of variables: 1) Baseline demographics, including age, sex, and baseline BMI, 2) Baseline Eastern cooper- active oncology group (ECOG) performance score that describes patients' level of functioning in terms of their ability to care for themselves, daily activity and physical ability, and 3) Baseline cancer characteristics, including clinical T stage, lymph node involvement, histologic status, and Kirsten rat sarcoma virus (KRAS) biomarker status.

1.2 CCHS

For the cardiovascular health dataset, the outcome considered was the binary variable for CVH status and the primary exposure of interest was gender. The model included other relevant predictors (age, education, household income, household size, and whether the participant is a new immigrant or not) which were selected based on previous studies [5].

Cardiovascular diseases (CVD) continue to represent the leading cause of mortality and morbidity amongst women and men worldwide [6]. Biological differences between the sexes such as anatomical and physiological variations in coronary arteries and autonomic nervous system, alter the development and progression of CVD [7]. However, environment and lifestyle [8] as well as individuals' identity, roles, and relations in society may play an important role. These characteristics are gendered in the way that

they affect males and females differently and evolve through early life to adulthood [9]. The specific model we evaluated is a classification version of the regression model predicting CVH [10].

1.3 DCCG

This is a prospectively maintained Danish Colorectal Cancer Group (DCCG) database including all Danish patients with a first-time diagnosis of right-sided colonic cancer between 2001 and 2018 [11]. The main outcome that we model is medical complications after surgery. The covariate of interest is sex.

The literature about post-operative outcomes in colon surgery shows different results regarding the effect of gender on post-operative complications. However, a snapshot prospective audit conducted by the European Society of Colo-Proctology (ESCP) provided real-time international data [12]. The study showed higher rates of post-operative complications in men (OR 1.5 CI 95% [1.2-1.8], $p<0.001$) who underwent right-sided colon resection for colon cancer. This has been confirmed by another snapshot prospective audit on left colon resection [13] which reported higher rate of post-operative complications in men (OR 1.46 CI 95% [1.16–1.84], p= 0.001). It is also interesting to see that the rate of conversion from laparoscopic to open surgery is higher in male gender (OR 1.50 CI 95% [1.17–1.93], p= 0.001). The hormonal effect of female hormones might have a protective effect as shown by lower rates of post-operative infection after elective colorectal surgery [14]. Despite these findings, men take shorter time to physically recover after colorectal surgery[15]. The reasons of disparity in outcome between men and women need to be investigated further.

The other covariates included in the model were: Age, ASA score, Localization of tumor, Procedure, Pathological T stage, Pathological N stage, Pathologically shown total number of removed lymph nodes, Pathologically shown total number of lymph nodes with metastasis, and Unplanned intra-operative adverse event (UIAEs).

2. Additional Results Figures for the Sequential Synthesis Generative Model

2.1 DCCG Results

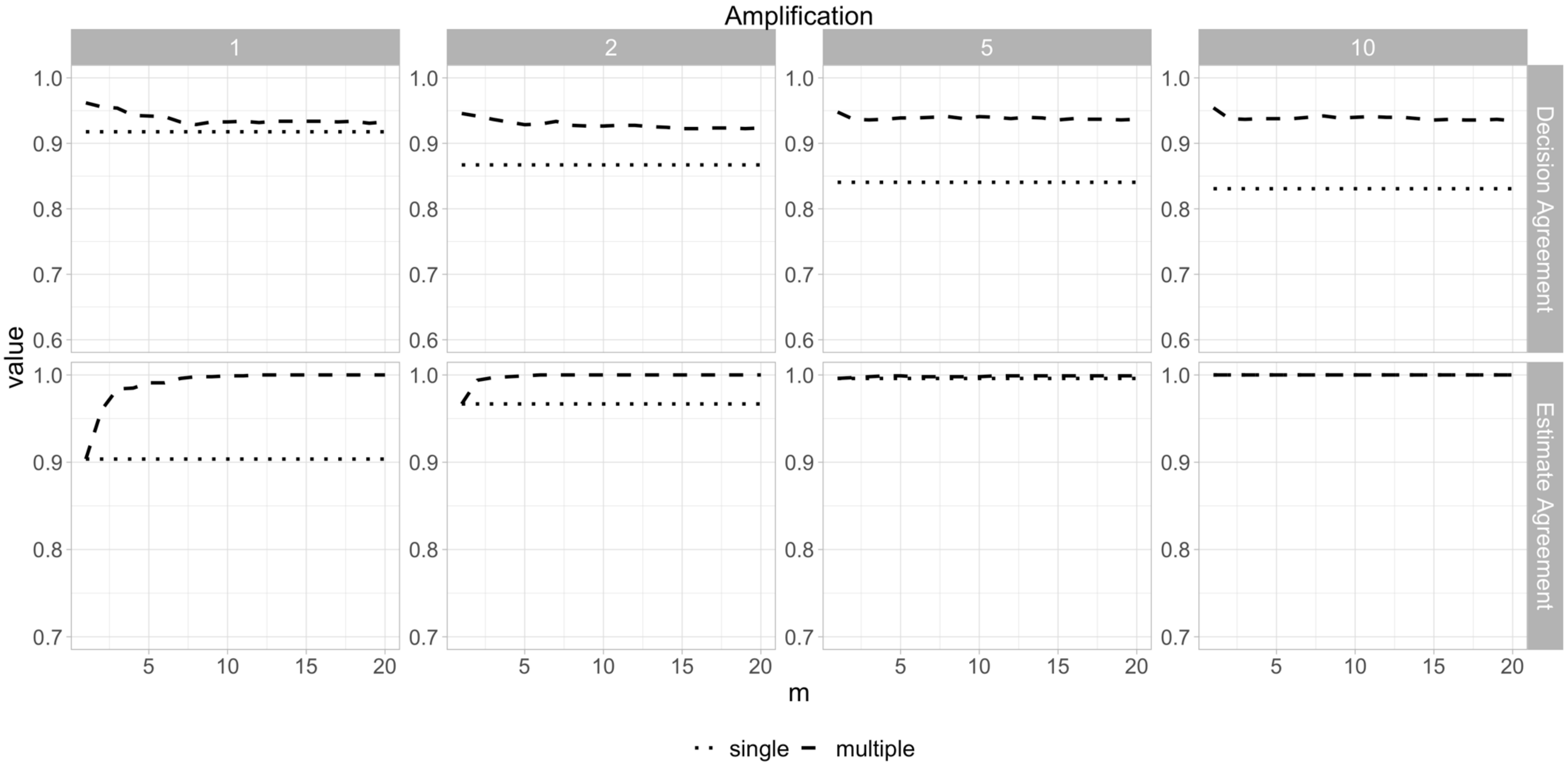

Figure 1: Decision agreement and estimate agreement for the **DCCG** colon cancer dataset using the sequential synthesis method.

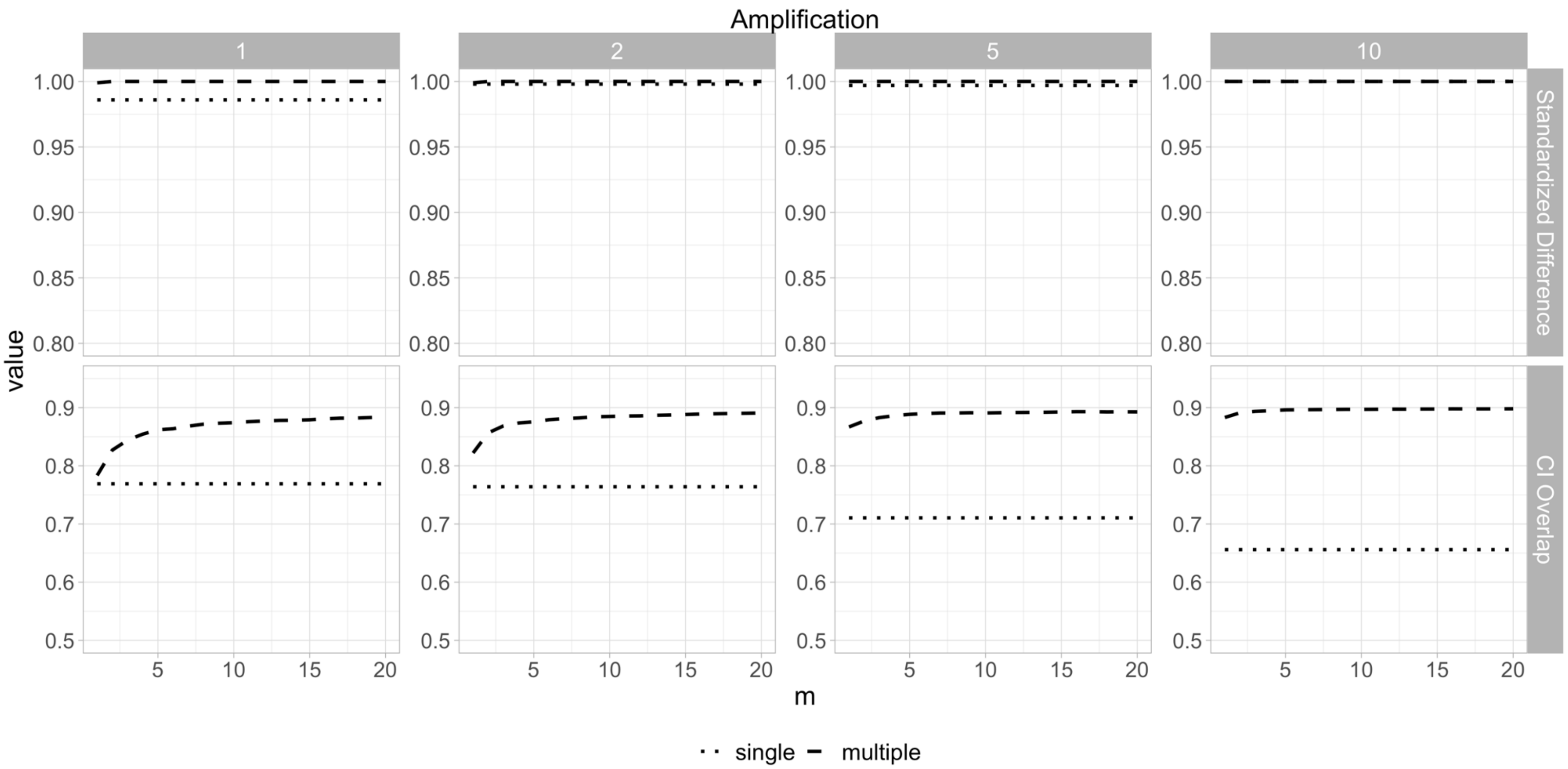

Figure 2: Standardized difference and confidence interval overlap for the **DCCG** colon cancer dataset using the sequential synthesis method.

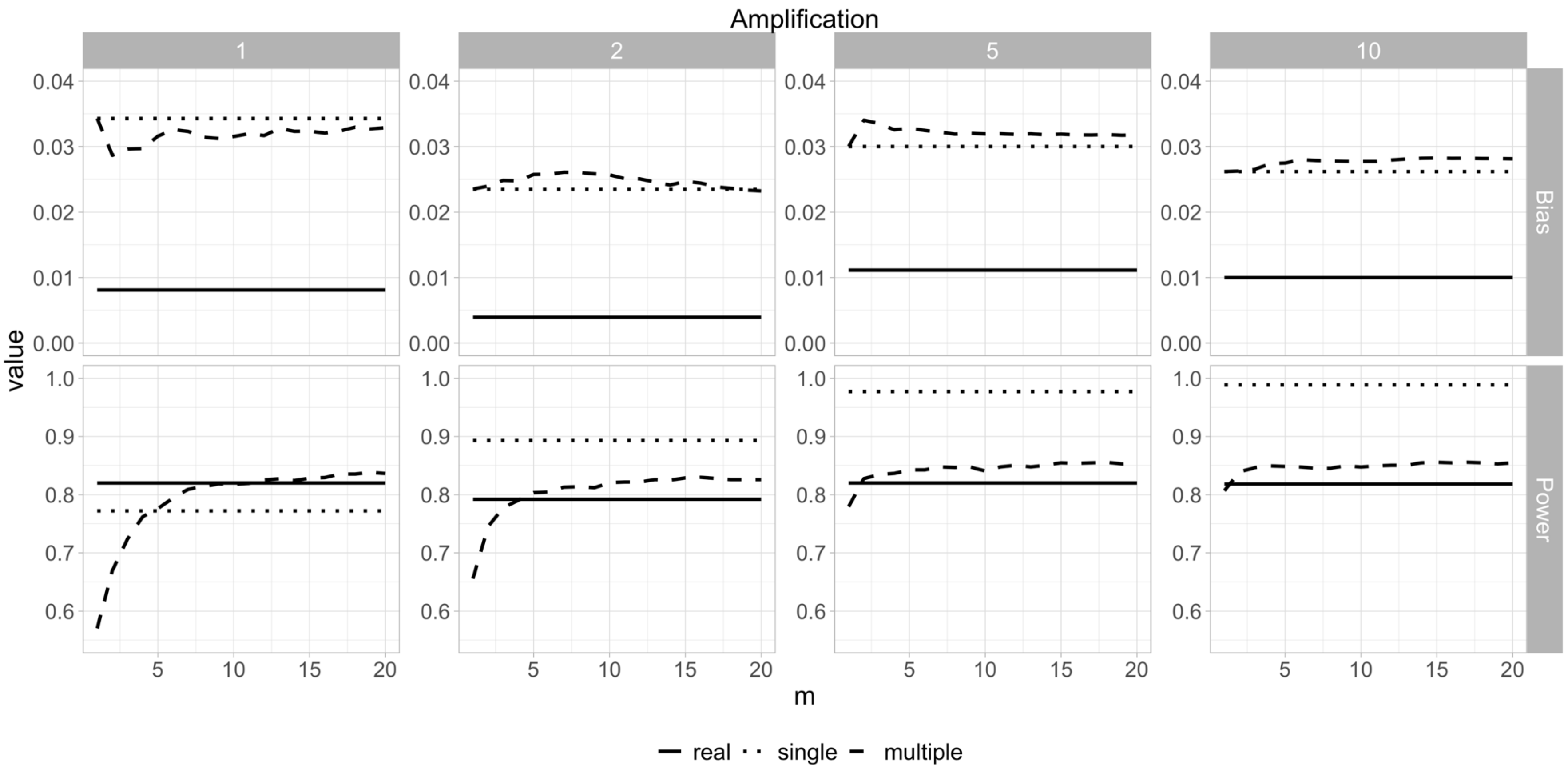

Figure 3: The bias and power for the **Danish (DCCG)** colon cancer breast cancer dataset using the sequential synthesis method.

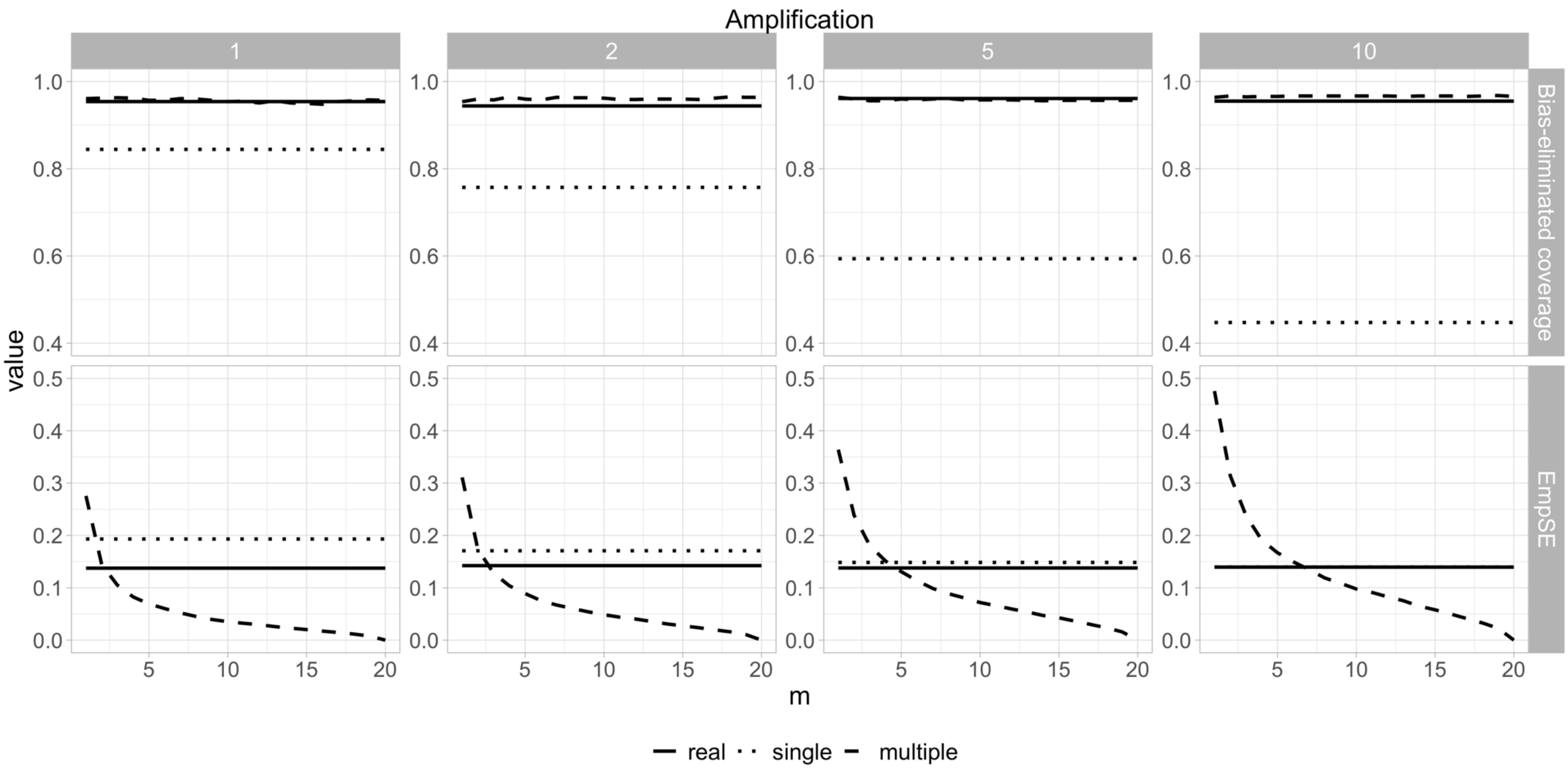

Figure 4: The coverage and empirical SE for the **Danish (DCCG)** colon cancer breast cancer dataset using the sequential synthesis method.

2.2 CCHS Results

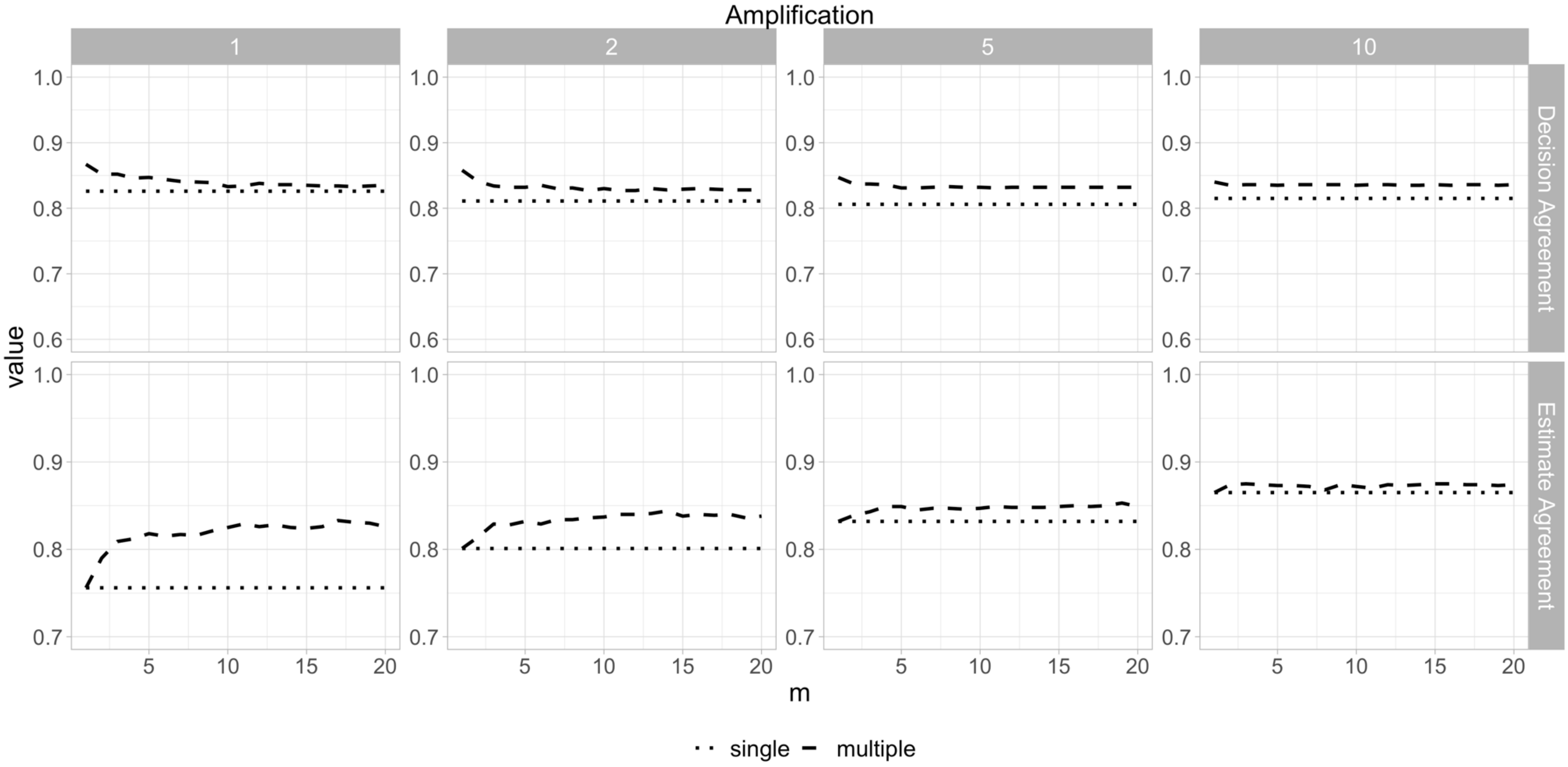

Figure 5: Decision agreement and estimate agreement for the **CCHS** dataset using the sequential synthesis method.

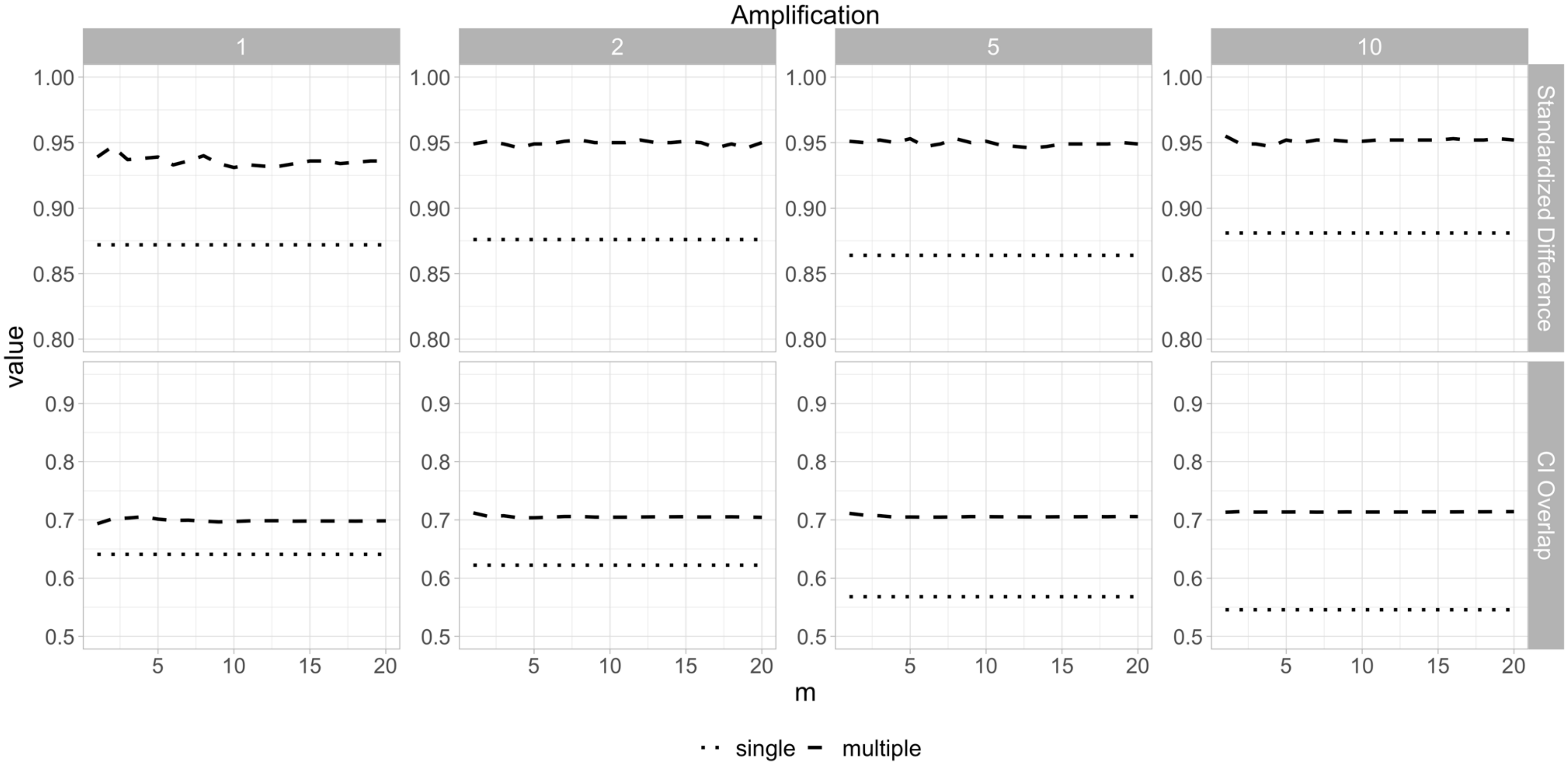

Figure 6: Standardized difference and confidence interval overlap for the **CCHS** dataset using the sequential synthesis method.

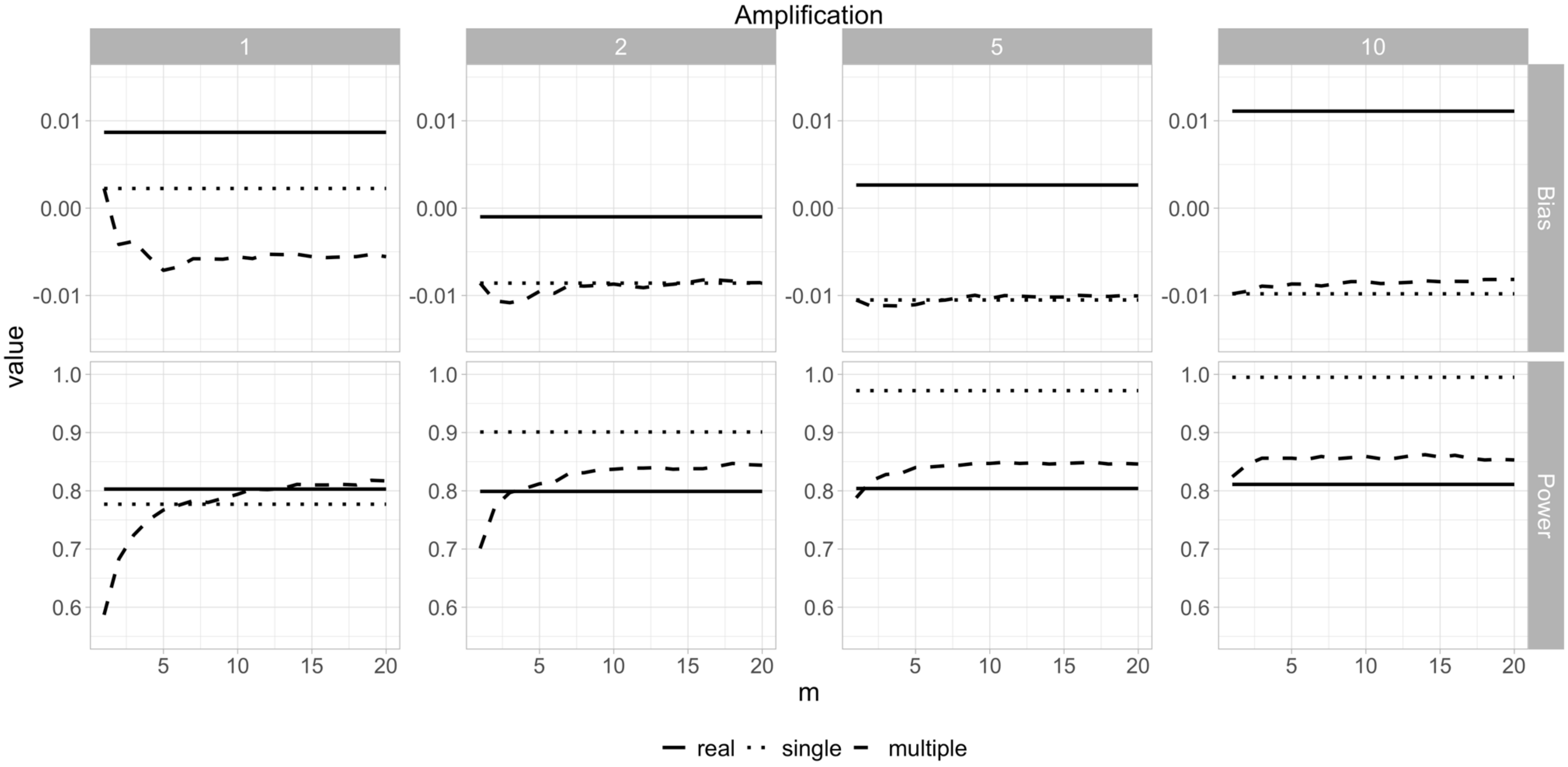

Figure 7: The bias and power for the **CCHS** dataset using the sequential synthesis method.

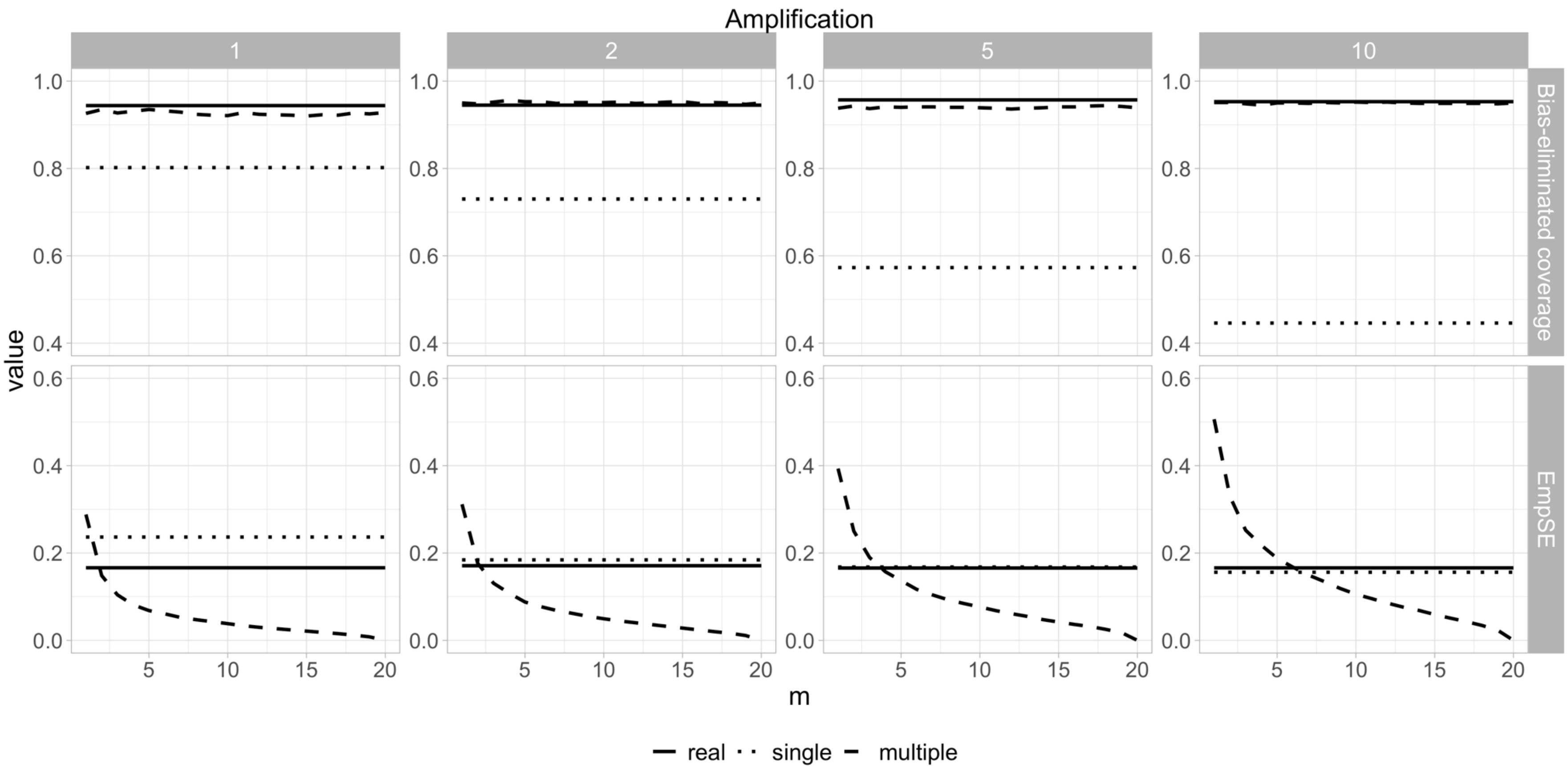

Figure 8: The coverage and empirical SE for the **CCHS** dataset using the sequential synthesis method.

3. Results Figures for the CTGAN Generative Model

3.1 N0147 Results

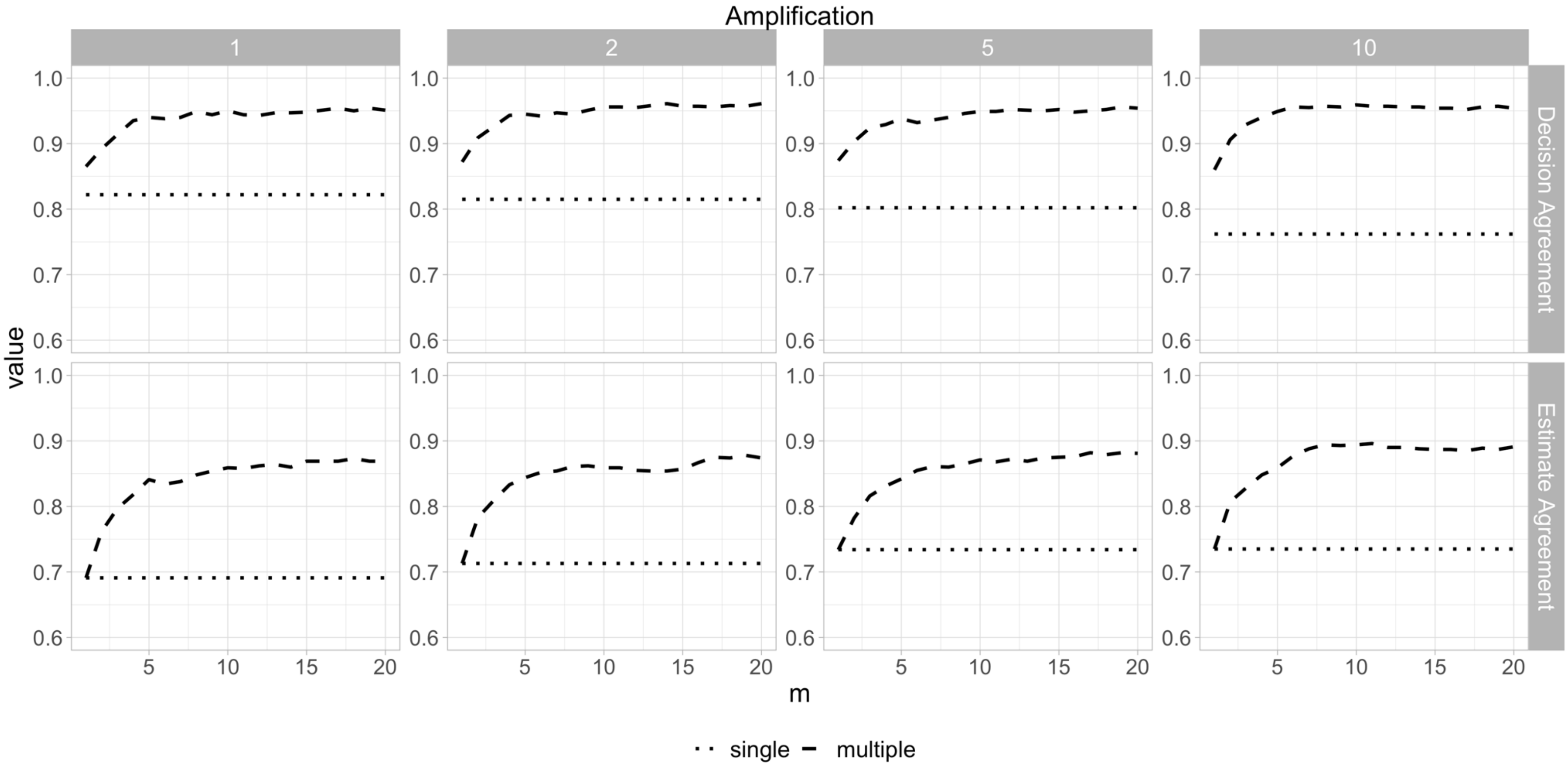

Figure 9: Decision agreement and estimate agreement for the **N0147** colon cancer dataset using the CTGAN method.

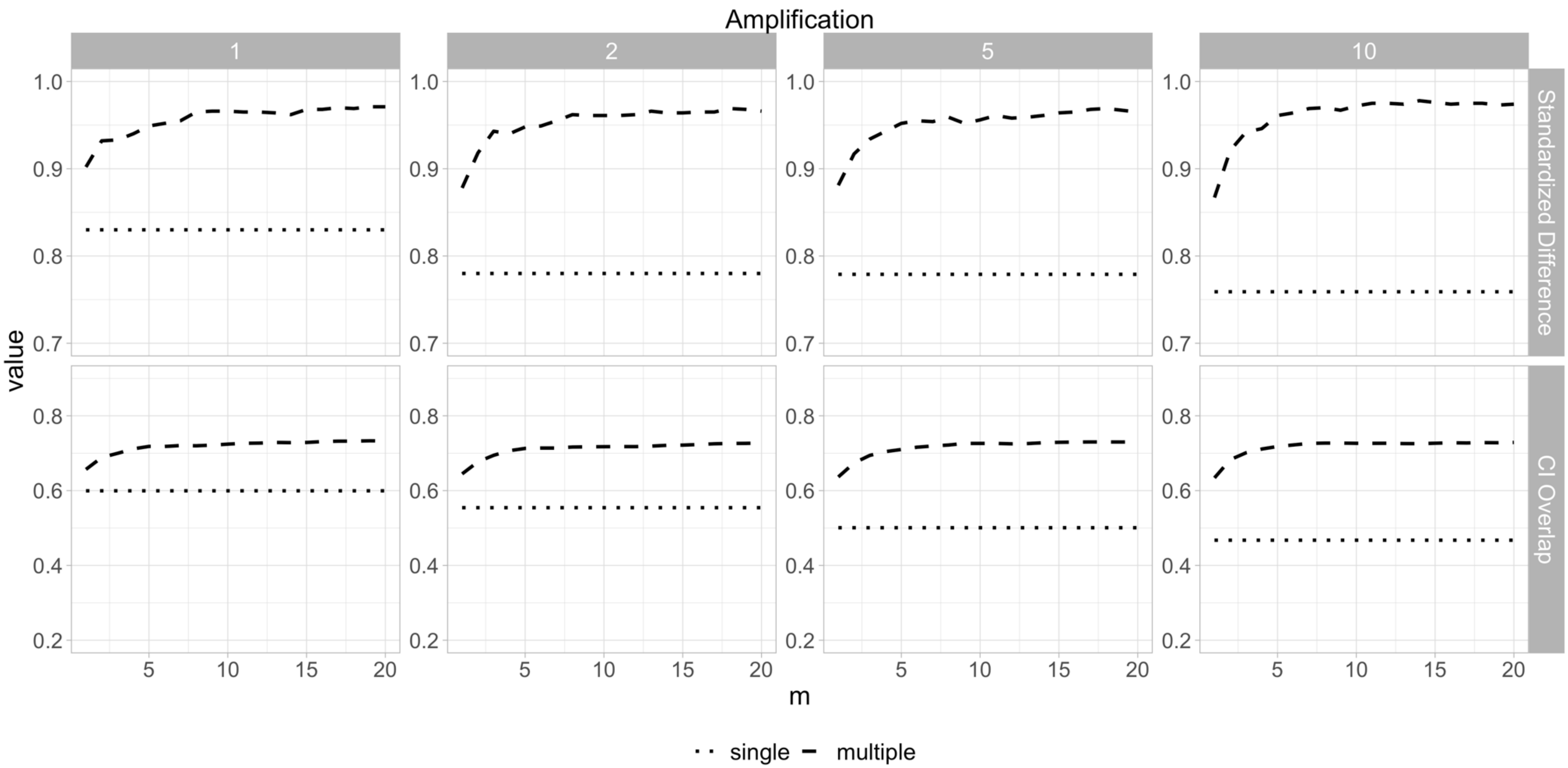

Figure 10: Standardized and confidence interval overlap for the **N0147** colon cancer dataset using the CTGAN method.

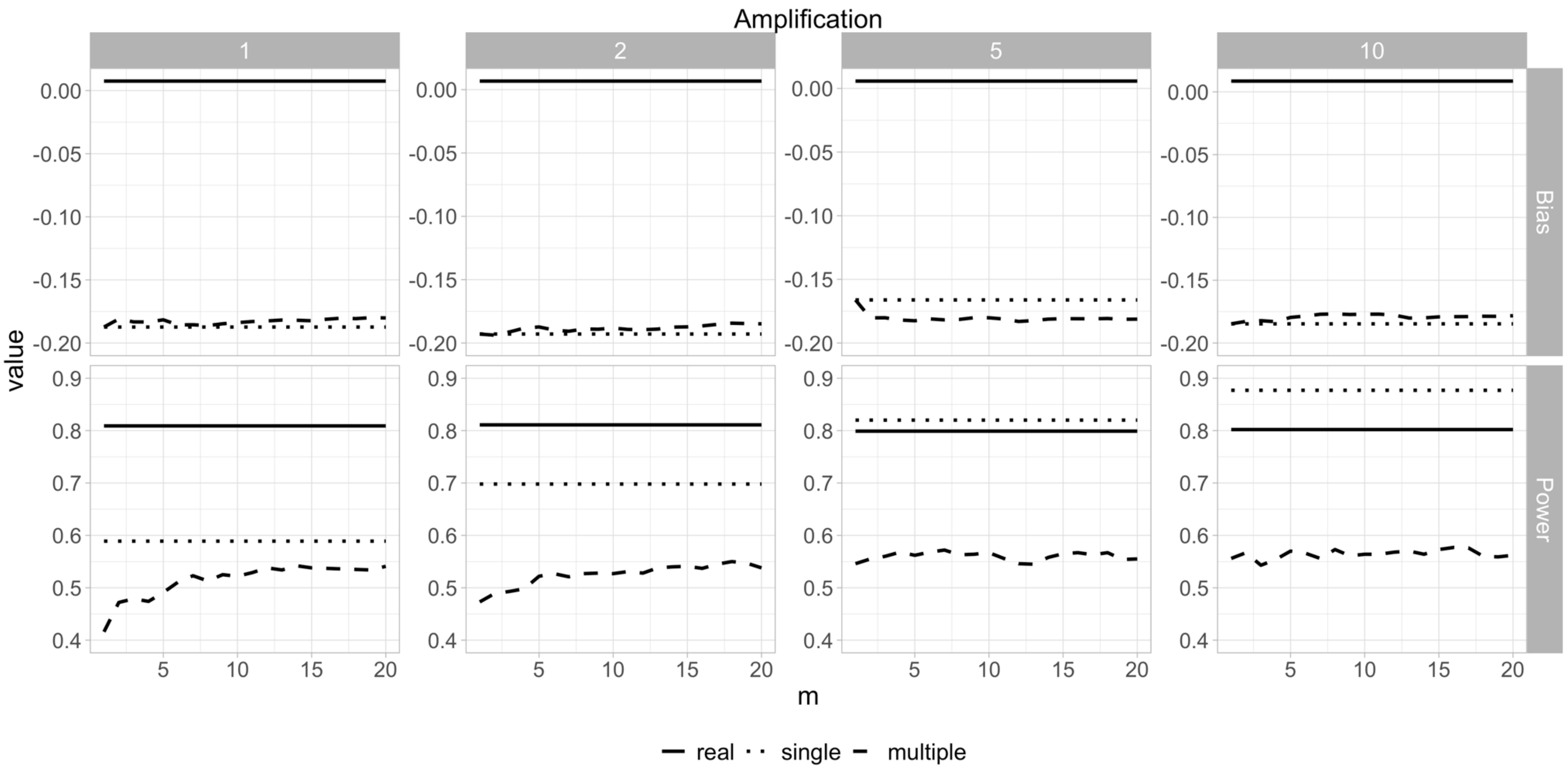

Figure 11: The bias and power for the **N0147** colon cancer dataset using CTGAN.

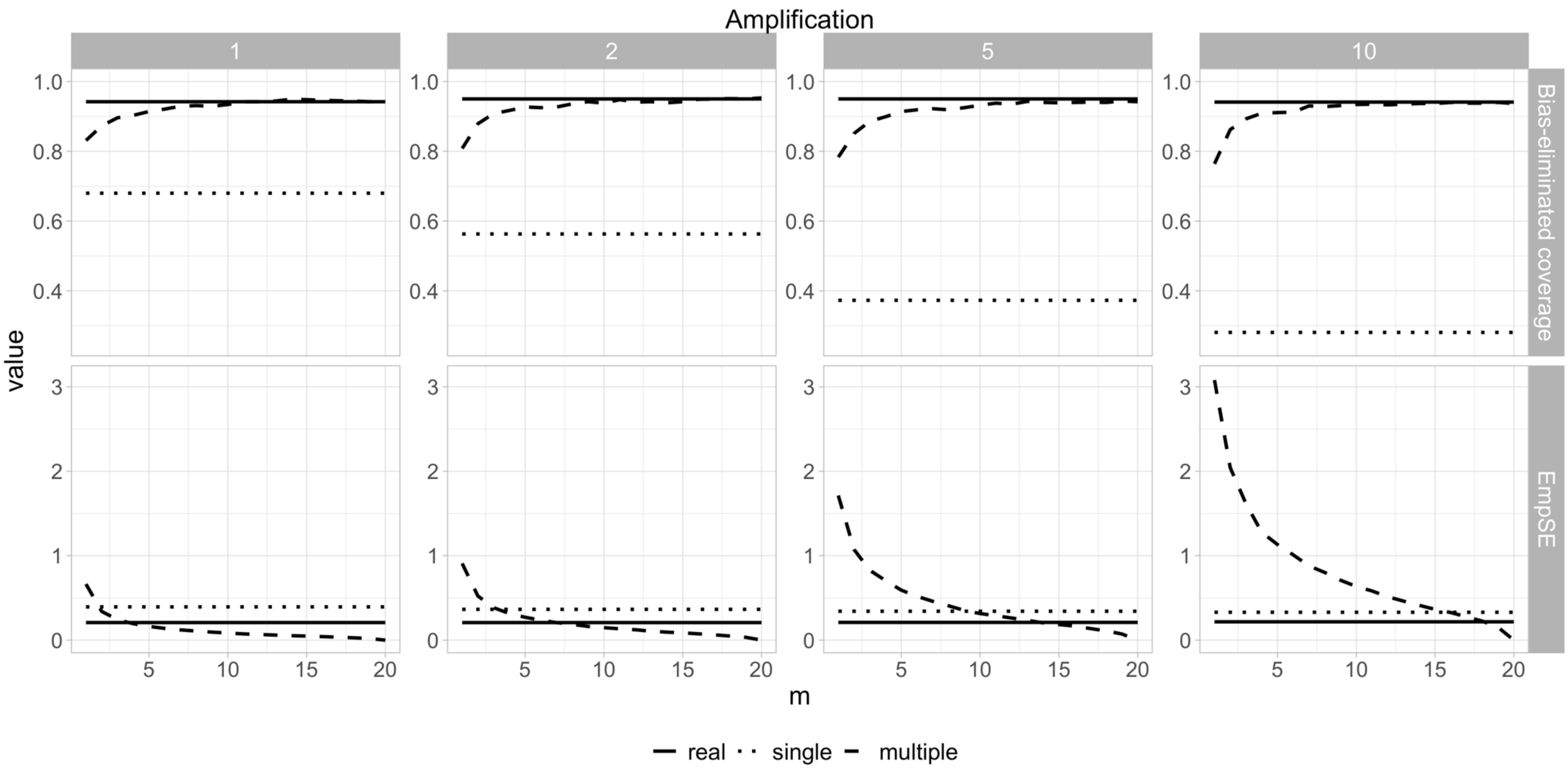

Figure 12: The coverage and empirical SE for the **N0147** colon cancer dataset using CTGAN.

3.2 DCCG Results

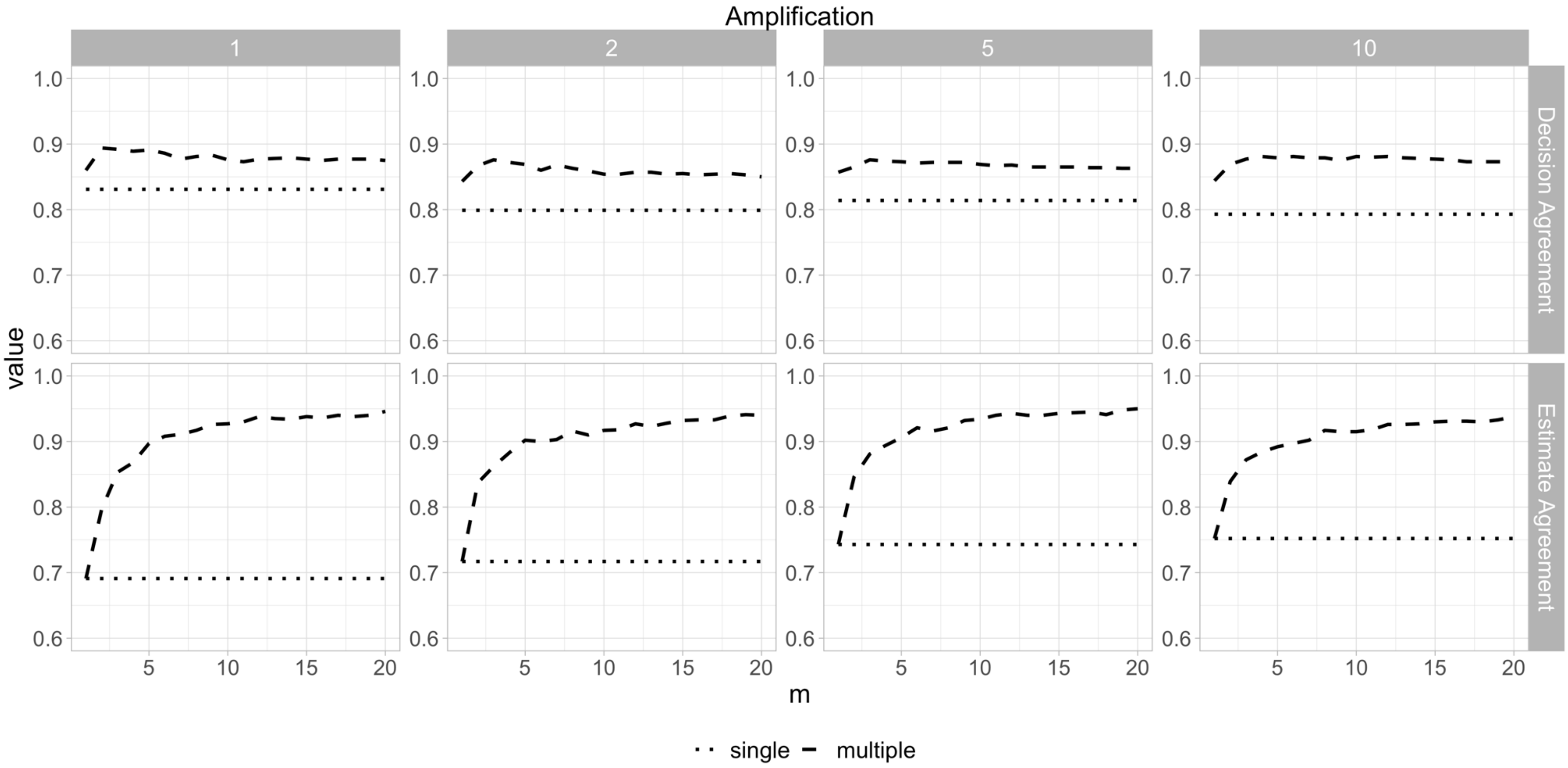

Figure 13: Decision agreement and estimate agreement for the **DCCG** colon cancer dataset using the CTGAN method.

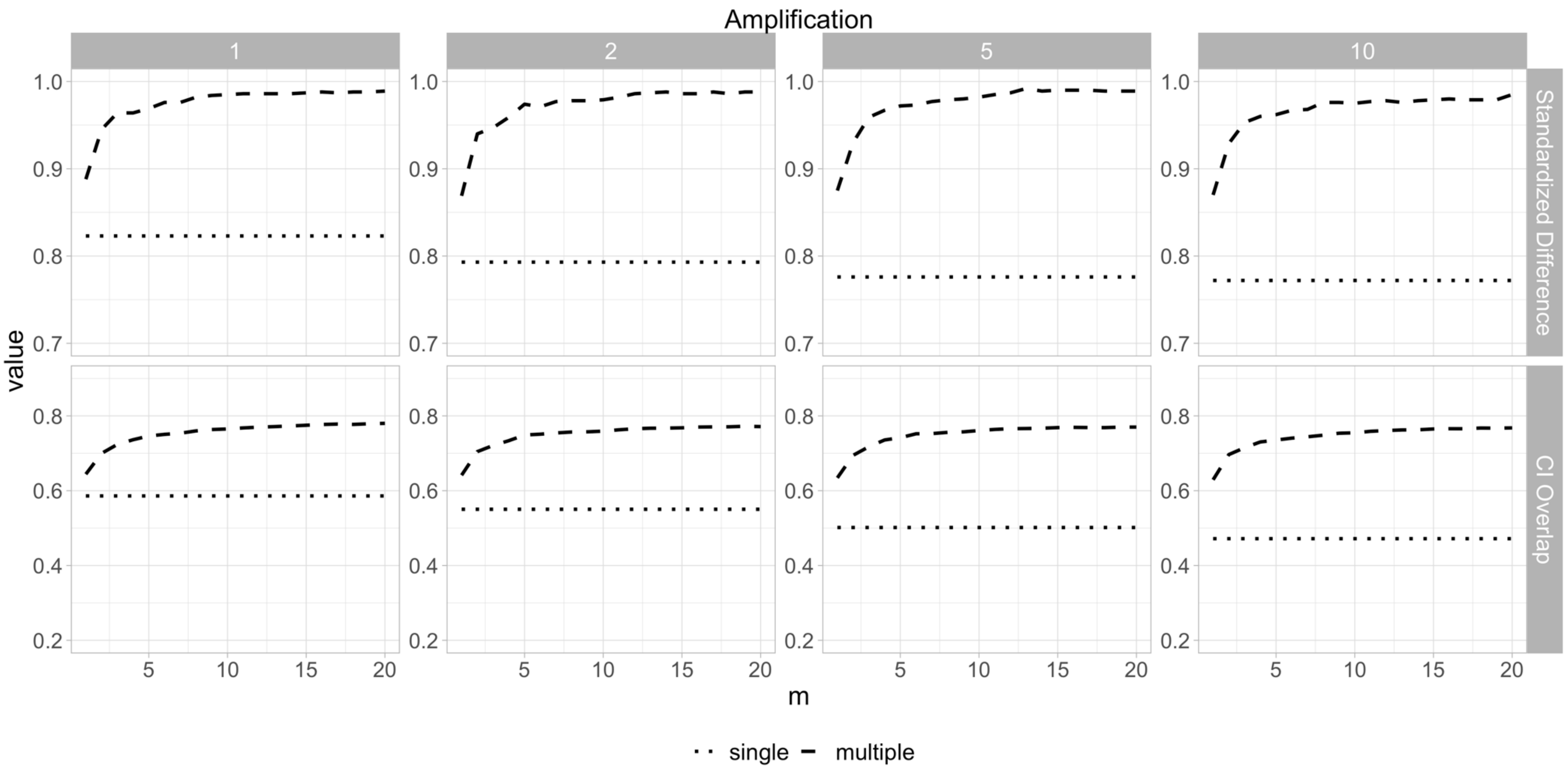

Figure 14: Standardized difference and confidence interval overlap for the **DCCG** colon cancer dataset using the CTGAN method.

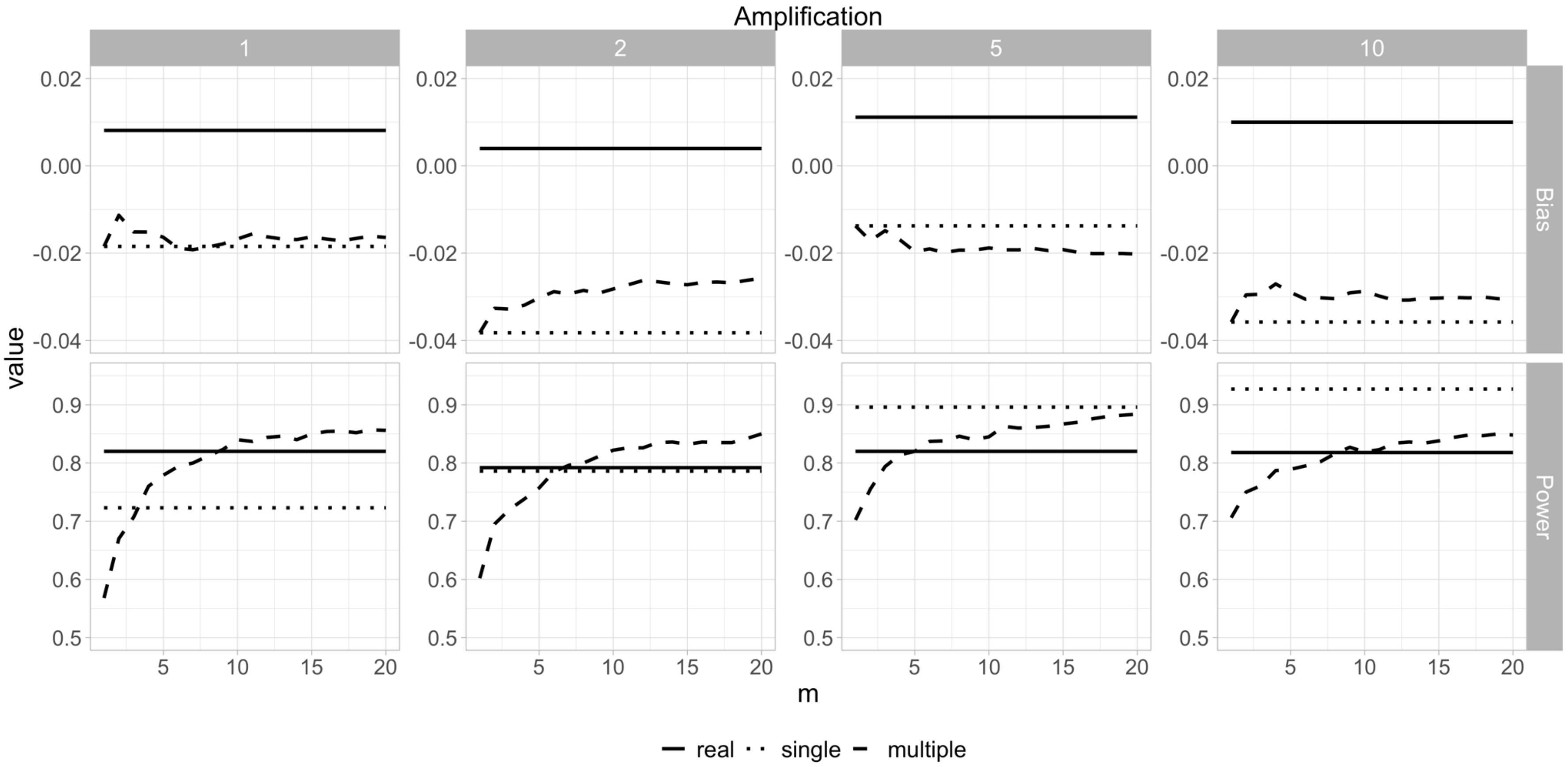

Figure 15: The bias and power for the **Danish (DCCG)** colon cancer dataset using CTGAN.

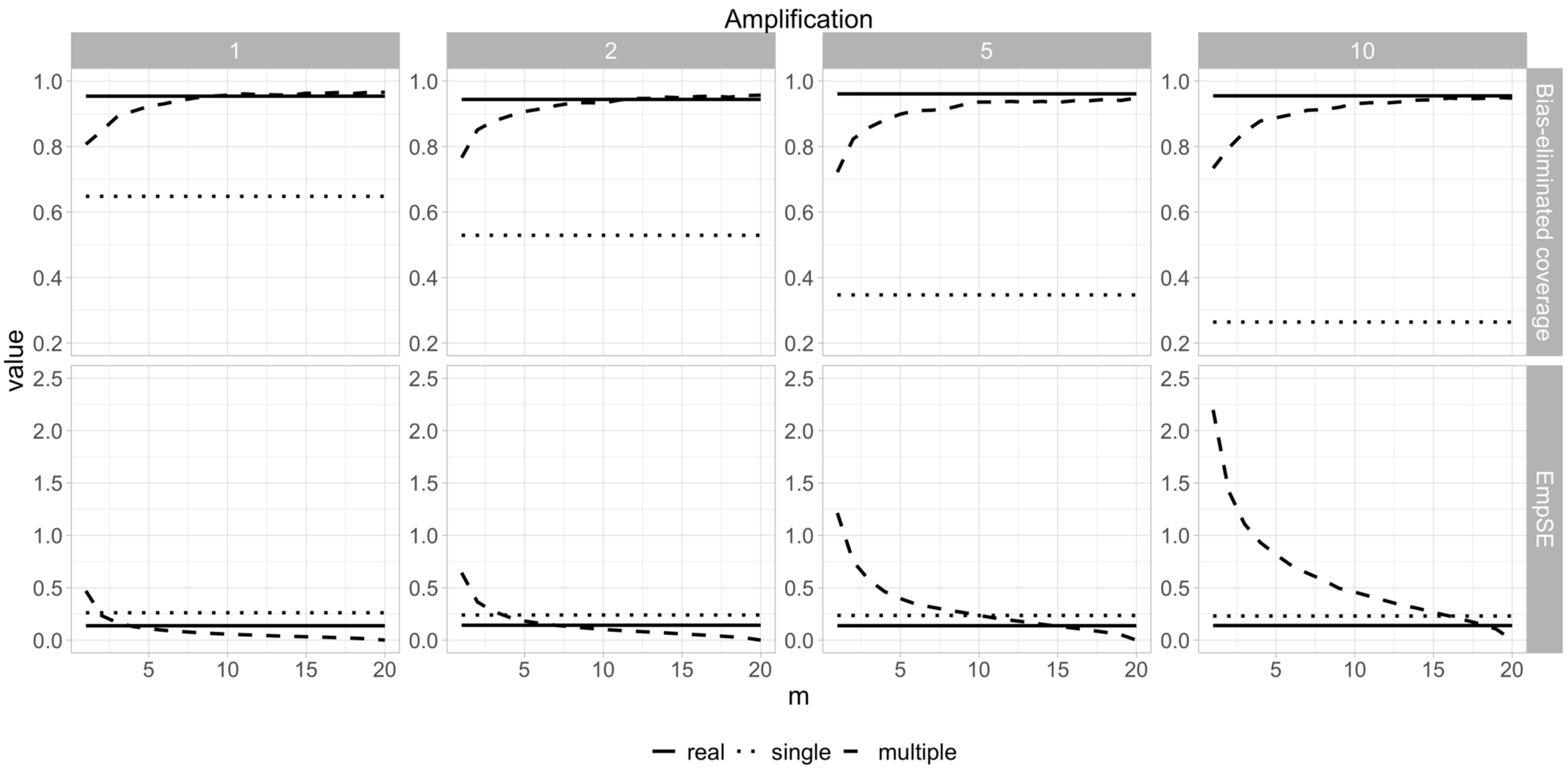

Figure 16: The coverage and empirical SE for the **Danish (DCCG)** colon cancer dataset using CTGAN.

3.3 CCHS Results

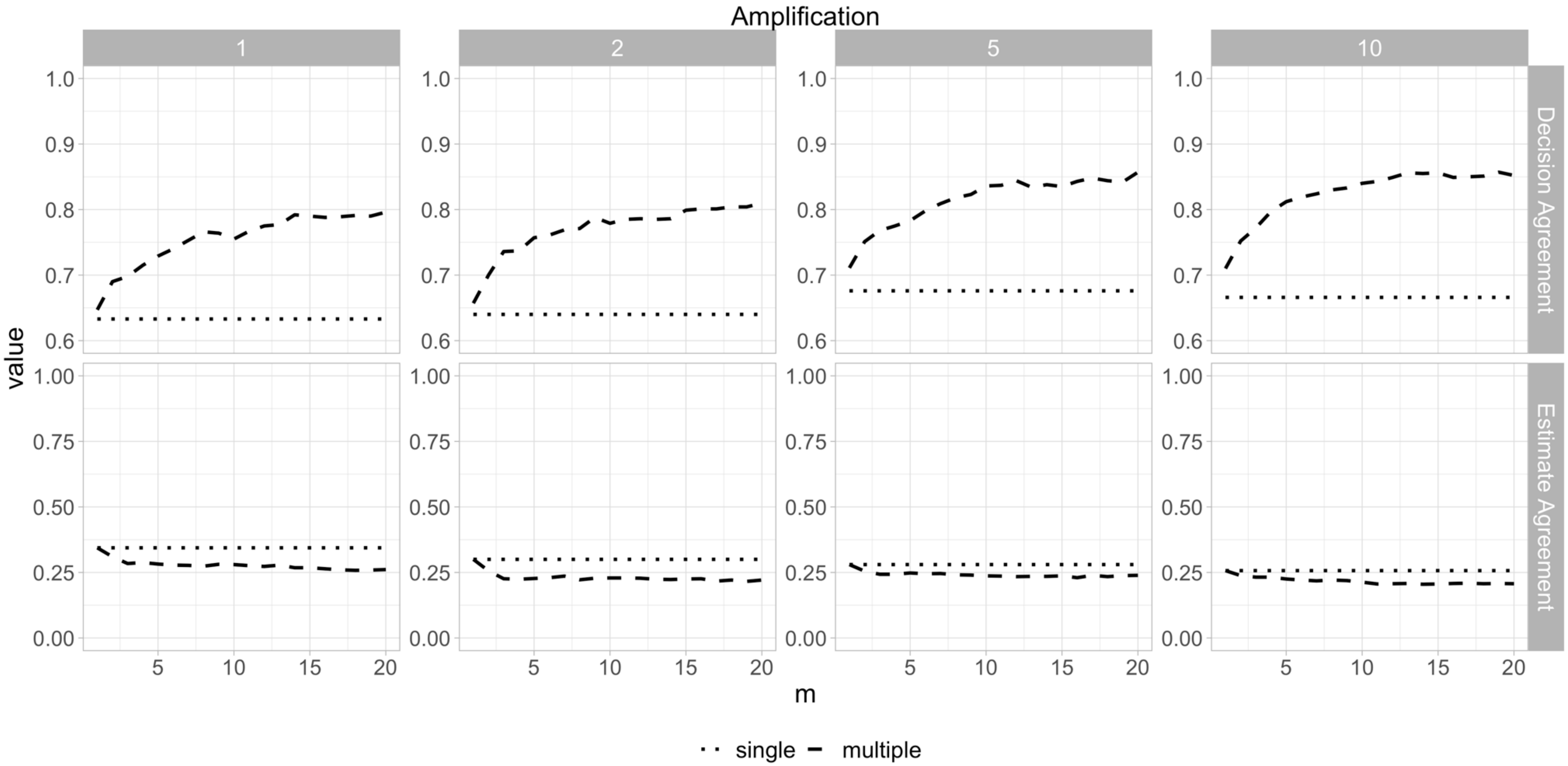

Figure 17: Decision agreement and estimate agreement for the **CCHS** dataset using the CTGAN method.

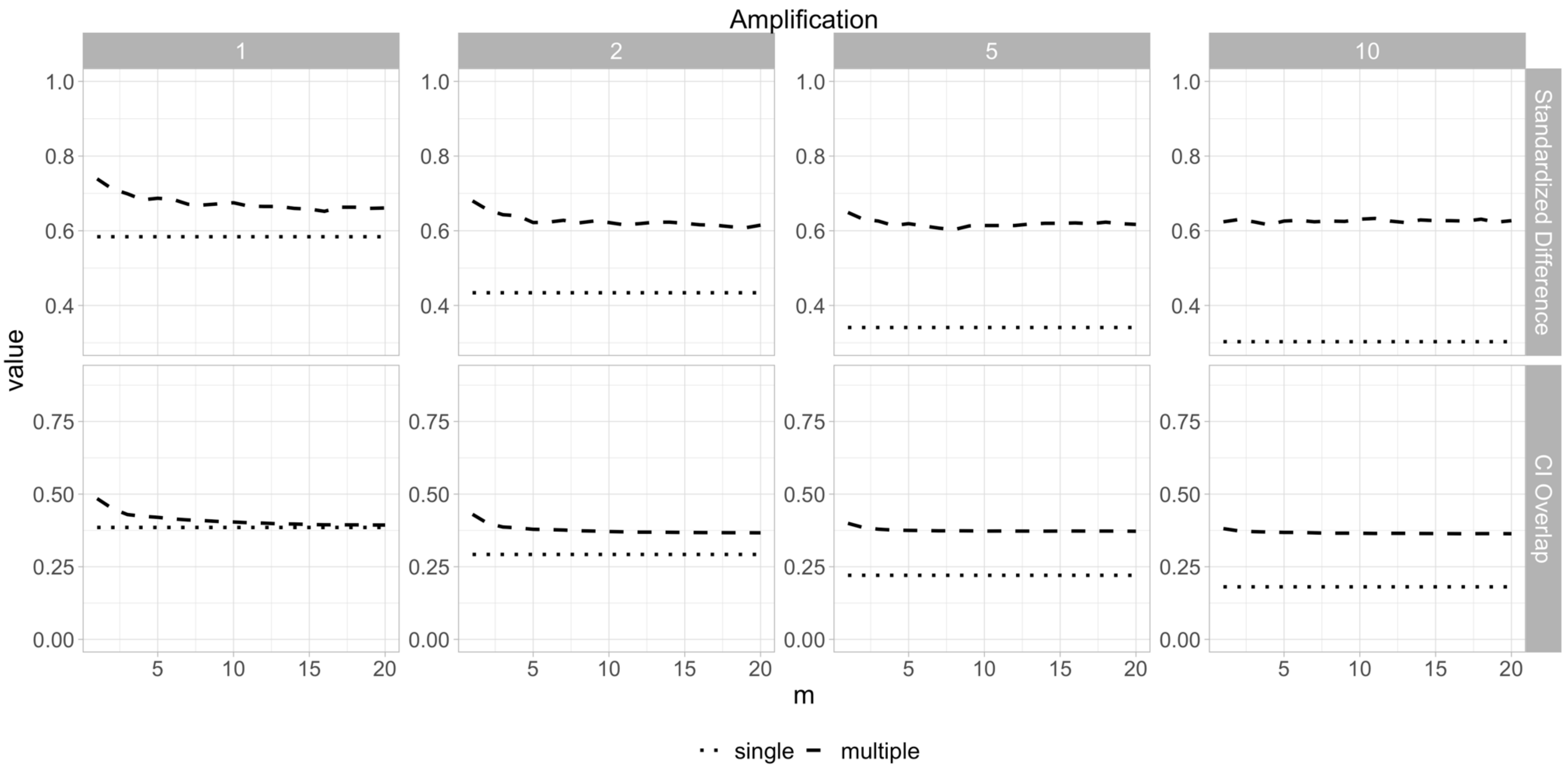

Figure 18: Standardized difference and confidence interval overlap for the **CCHS** dataset using the CTGAN method.

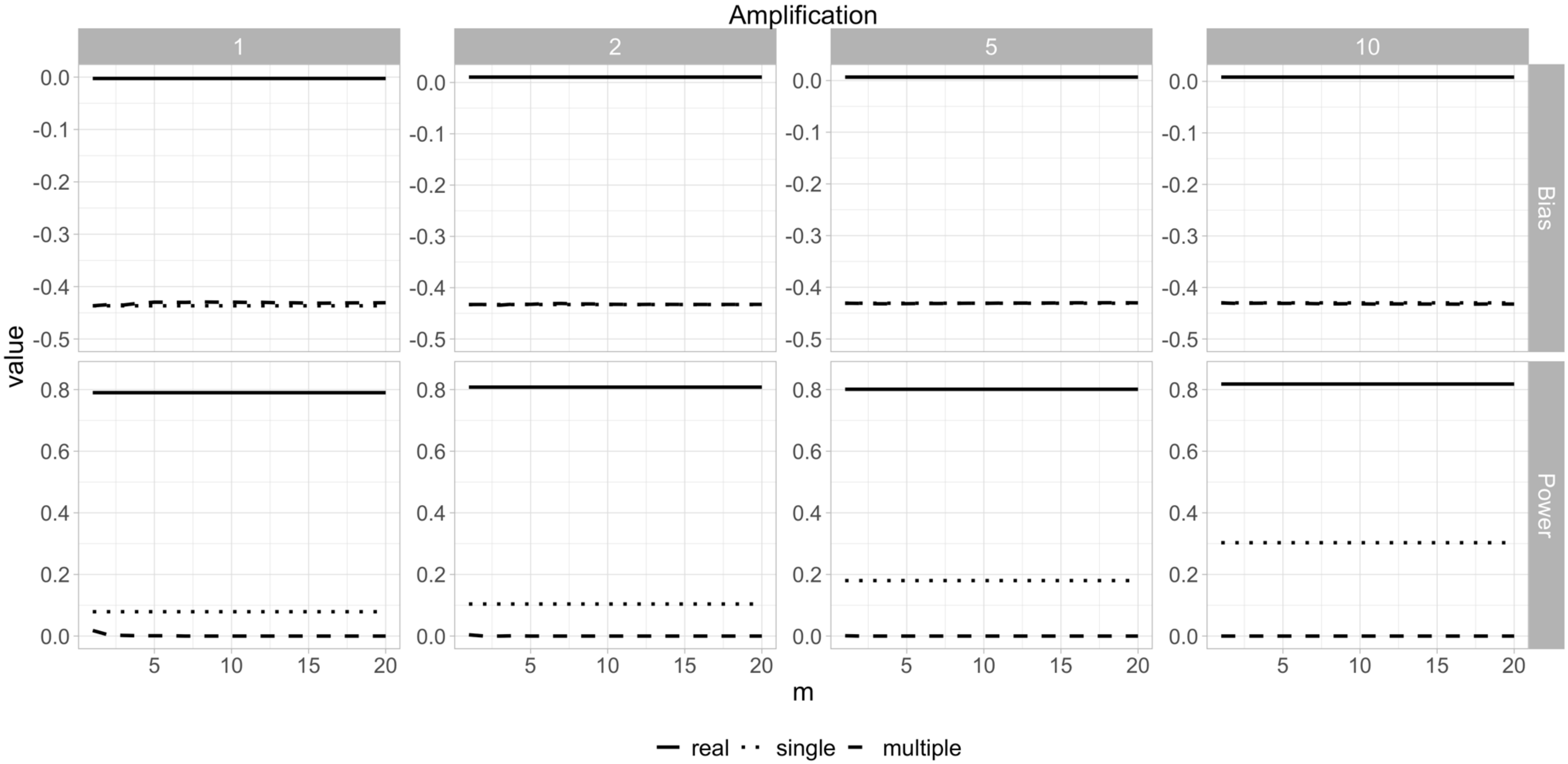

Figure 19: The bias and power for the **CCHS** dataset using CTGAN.

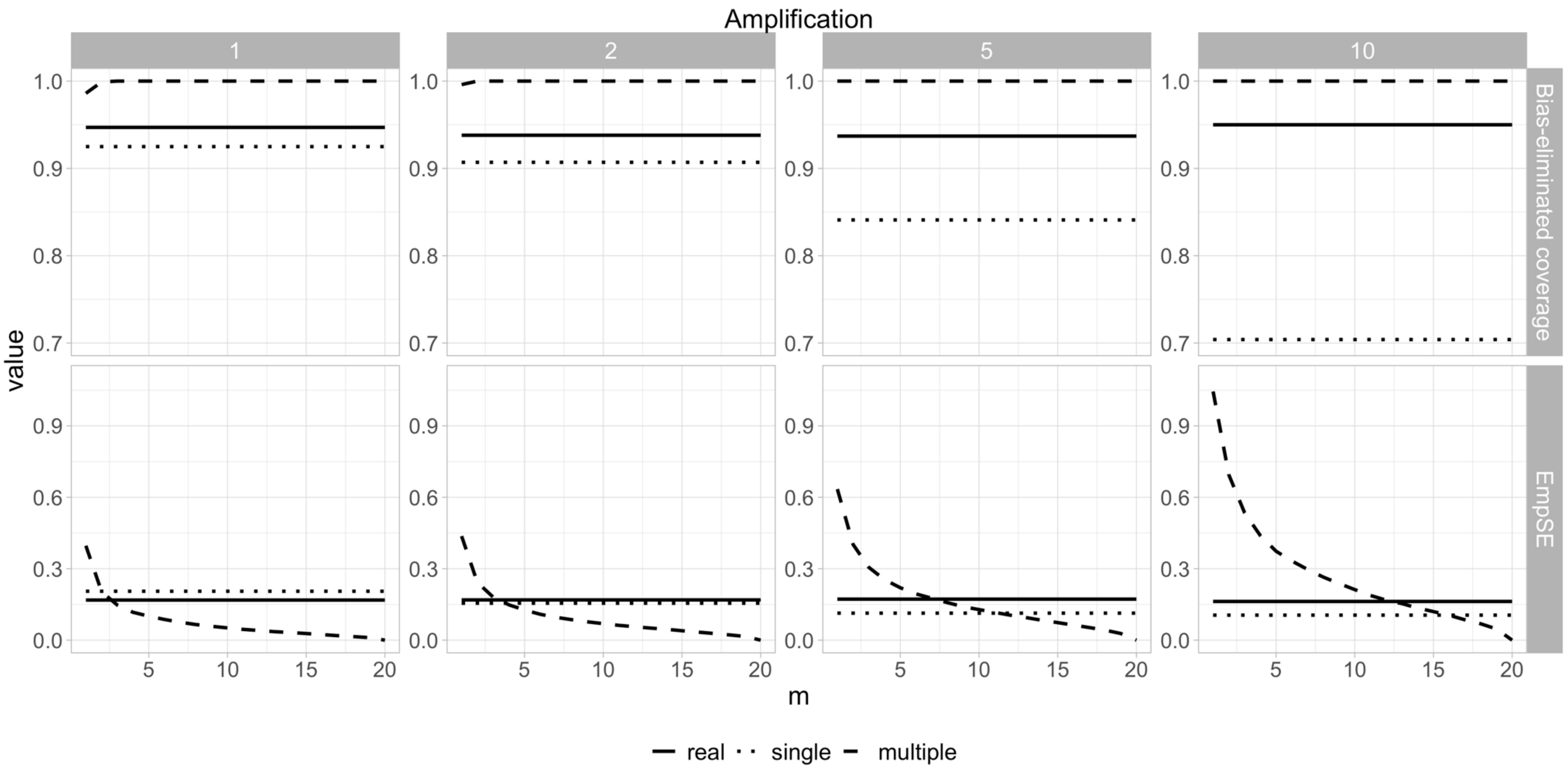

Figure 20: The coverage and empirical SE for the **CCHS** dataset using CTGAN.

3.4 Membership Disclosure Results

	Membership Disclosure
N0147	0.00493
DCCG	-0.0001
CCHS	0.01088

Table 1: Average membership disclosure values for the three datasets using the CTGAN generative model.

4. References

[1] S. R. Alberts *et al.*, “Effect of oxaliplatin, fluorouracil, and leucovorin with or without cetuximab on survival among patients with resected stage III colon cancer: a randomized trial,” *JAMA*, vol. 307, no. 13, pp. 1383–1393, Apr. 2012, doi: 10.1001/jama.2012.385.

[2] F. S. Dahdaleh *et al.*, “Obstruction predicts worse long-term outcomes in stage III colon cancer: A secondary analysis of the N0147 trial,” *Surgery*, vol. 164, no. 6, pp. 1223–1229, 2018, doi: 10.1016/j.surg.2018.06.044.

[3] P. G. Carraro, M. Segala, B. M. Cesana, and G. Tiberio, “Obstructing colonic cancer: failure and survival patterns over a ten-year follow-up after one-stage curative surgery,” *Dis. Colon Rectum*, vol. 44, no. 2, pp. 243–250, Feb. 2001, doi: 10.1007/BF02234300.

[4] J. Mella, A. Biffin, A. G. Radcliffe, J. D. Stamatakis, and R. J. Steele, “Population-based audit of colorectal cancer management in two UK health regions. Colorectal Cancer Working Group, Royal College of Surgeons of England Clinical Epidemiology and Audit Unit,” *Br J Surg*, vol. 84, no. 12, pp. 1731–1736, Dec. 1997.

[5] K. E. Emam, L. Mosquera, X. Fang, and A. El-Hussuna, “Utility Metrics for Evaluating Synthetic Health Data Generation Methods: Validation Study,” *JMIR Medical Informatics*, vol. 10, no. 4, p. e35734, Apr. 2022, doi: 10.2196/35734.

[6] S. S. Virani *et al.*, “Heart Disease and Stroke Statistics-2020 Update: A Report From the American Heart Association,” *Circulation*, vol. 141, no. 9, pp. e139–e596, Mar. 2020, doi: 10.1161/CIR.0000000000000757.

[7] V. H. Huxley, “Sex and the cardiovascular system: the intriguing tale of how women and men regulate cardiovascular function differently,” *Adv Physiol Educ*, vol. 31, no. 1, pp. 17–22, Mar. 2007, doi: 10.1152/advan.00099.2006.

[8] P. J. Connelly, Z. Azizi, P. Alipour, C. Delles, L. Pilote, and V. Raparelli, “The Importance of Gender to Understand Sex Differences in Cardiovascular Disease,” *Can J Cardiol*, vol. 37, no. 5, pp. 699–710, May 2021, doi: 10.1016/j.cjca.2021.02.005.

[9] D. Bartz *et al.*, “Clinical Advances in Sex- and Gender-Informed Medicine to Improve the Health of All: A Review,” *JAMA Intern Med*, vol. 180, no. 4, pp. 574–583, Apr. 2020, doi: 10.1001/jamainternmed.2019.7194.

[10] Z. Azizi *et al.*, “A comparison of synthetic data generation and federated analysis for enabling international evaluations of cardiovascular health,” *Sci Rep*, vol. 13, no. 1, Art. no. 1, Jul. 2023, doi: 10.1038/s41598-023-38457-3.

[11] A. El-Hussuna, T. Lytras, N. H. Bruun, M. F. Klein, S. H. Emile, and N. Qvist, “Extended Right-Sided Colon Resection Does Not Reduce the Risk of Colon Cancer Local-Regional Recurrence: Nationwide

Population-Based Study from Danish Colorectal Cancer Group Database," *Diseases of the Colon & Rectum*, pp. 10–1097, 2022, doi: https://doi.org/10.1097/DCR.0000000000002358.
[12] 2015 European Society of Coloproctology Collaborating Group, "Predictors for Anastomotic Leak, Postoperative Complications, and Mortality After Right Colectomy for Cancer: Results From an International Snapshot Audit," *Dis Colon Rectum*, vol. 63, no. 5, pp. 606–618, May 2020, doi: 10.1097/DCR.0000000000001590.
[13] 2017 and 2015 European Society of Coloproctology (ESCP) collaborating groups, "The impact of conversion on the risk of major complication following laparoscopic colonic surgery: an international, multicentre prospective audit," *Colorectal Dis*, vol. 20 Suppl 6, pp. 69–89, Sep. 2018, doi: 10.1111/codi.14371.
[14] D. N. Blitzer, J. M. Davis, N. Ahmed, Y.-H. Kuo, and Y.-L. Kuo, "Impact of procedure on the post-operative infection risk of patients after elective colon surgery," *Surg Infect (Larchmt)*, vol. 15, no. 6, pp. 721–725, Dec. 2014, doi: 10.1089/sur.2013.147.
[15] E. McCaughan, G. Prue, K. Parahoo, S. McIlfatrick, and H. McKenna, "Exploring and comparing the experience and coping behaviour of men and women with colorectal cancer after chemotherapy treatment: a qualitative longitudinal study," *Psycho-Oncology*, vol. 21, no. 1, pp. 64–71, 2012, doi: 10.1002/pon.1871.
